# Identification of thioridazine, an antipsychotic drug, as an antiglioblastoma and anticancer stem cell agent using public gene expression data

**DOI:** 10.1038/cddis.2015.77

**Published:** 2015-05-07

**Authors:** H-W Cheng, Y-H Liang, Y-L Kuo, C-P Chuu, C-Y Lin, M-H Lee, A T H Wu, C-T Yeh, E I-T Chen, J Whang-Peng, C-L Su, C-YF Huang

**Affiliations:** 1 Institute of Biopharmaceutical Sciences, National Yang-Ming University, Taipei, Taiwan; 2 Department of Biotechnology and Laboratory Science in Medicine, National Yang-Ming University, Taipei, Taiwan; 3 Department of Computer Science and Information Engineering, National Taiwan University, Taipei, Taiwan; 4 Institute of Cellular and System Medicine, National Health Research Institutes, Miaoli, Taiwan; 5 Institute of Cancer Research, National Health Research Institutes, Miaoli, Taiwan; 6 Department of Radiation, School of Medicine, Taipei Medical University, Taipei, Taiwan; 7 Graduate Institute of Clinical Medicine, Taipei Medical University, Taipei, Taiwan; 8 Municipal Wan Fang Hospital, Taipei Medical University, Taipei, Taiwan; 9 Program of Nutritional Science and Education, Department of Human Development and Family Studies, National Taiwan Normal University, Taipei, Taiwan

## Abstract

Glioblastoma (GBM) is a common and malignant tumor with a poor prognosis. Glioblastoma stem cells (GSCs) have been reported to be involved in tumorigenesis, tumor maintenance and therapeutic resistance. Thus, to discover novel candidate therapeutic drugs for anti-GBM and anti-GSCs is an urgent need. We hypothesized that if treatment with a drug could reverse, at least in part, the gene expression signature of GBM and GSCs, this drug may have the potential to inhibit pathways essential in the formation of GBM and thereby treat GBM. Here, we collected 356 GBM gene signatures from public databases and queried the Connectivity Map. We systematically evaluated the *in vitro* antitumor effects of 79 drugs in GBM cell lines. Of the drugs screened, thioridazine was selected for further characterization because it has potent anti-GBM and anti-GSCs properties. When investigating the mechanisms underlying the cytocidal effects of thioridazine, we found that thioridazine induces autophagy in GBM cell lines, and upregulates AMPK activity. Moreover, LC3-II was upregulated in U87MG sphere cells treated with thioridazine. In addition, thioridazine suppressed GBM tumorigenesis and induced autophagy *in vivo*. We not only repurposed the antipsychotic drug thioridazine as a potent anti-GBM and anti-GSCs agent, but also provided a new strategy to search for drugs with anticancer and anticancer stem cell properties.

Glioblastomas (GBM), the most common and most aggressive primary brain tumors in adults, are classified as grade IV astrocytomas by the World Health Organization and account for 54% of all gliomas.^1^ Surgery is typically followed by radiation therapy and chemotherapy with temozolomide (TMZ), which has been in clinical use since 2005.^2, 3^ Despite this multimodal approach, the median survival time of GBM patients is ~14.6 months.^3^ Therefore, a large number of new drugs are in development for GBM treatment.

Instead of focusing on a single drug target, using a batch of genes to query the Connectivity Map (Cmap, http://www.broad.mit.edu/cmap/) may not only allow multiple targets to be considered simultaneously, but it may also identify potential new drugs. Cmap is a database that provides ~7000 microarray expression profiles (conducted on Affymetrix HG-U133A arrays) from four different cancer cell lines treated with 1309 molecular drugs. Of the 1309 drugs included in Cmap, most are currently used in clinical treatment or are well-developed drugs; thus, we can rapidly identify potential drugs and proceed to clinical trial.

Thioridazine is an antipsychotic drug and is widely used to treat schizophrenia and psychosis. Recently, it has been shown that patients with schizophrenia have a lower risk of getting cancer (1.93%) than patients without schizophrenia (2.97%).^4^ In addition, inverse cancer comorbidity has been reported in people with certain CNS disorders, and pharmacological treatments is one of possible explanations.^5^

Using *in silico* drug screening via Cmap followed by empirical validations, we discovered that thioridazine can reduce the viability of GBM cells and GBM stem cells, induce autophagy and affect the expressions of related proteins in GBM cells. Thus, thioridazine has potential to treat GBM.

## Results

### Using GBM gene signatures to identify drugs for GBM via Cmap

**Figure 1:**
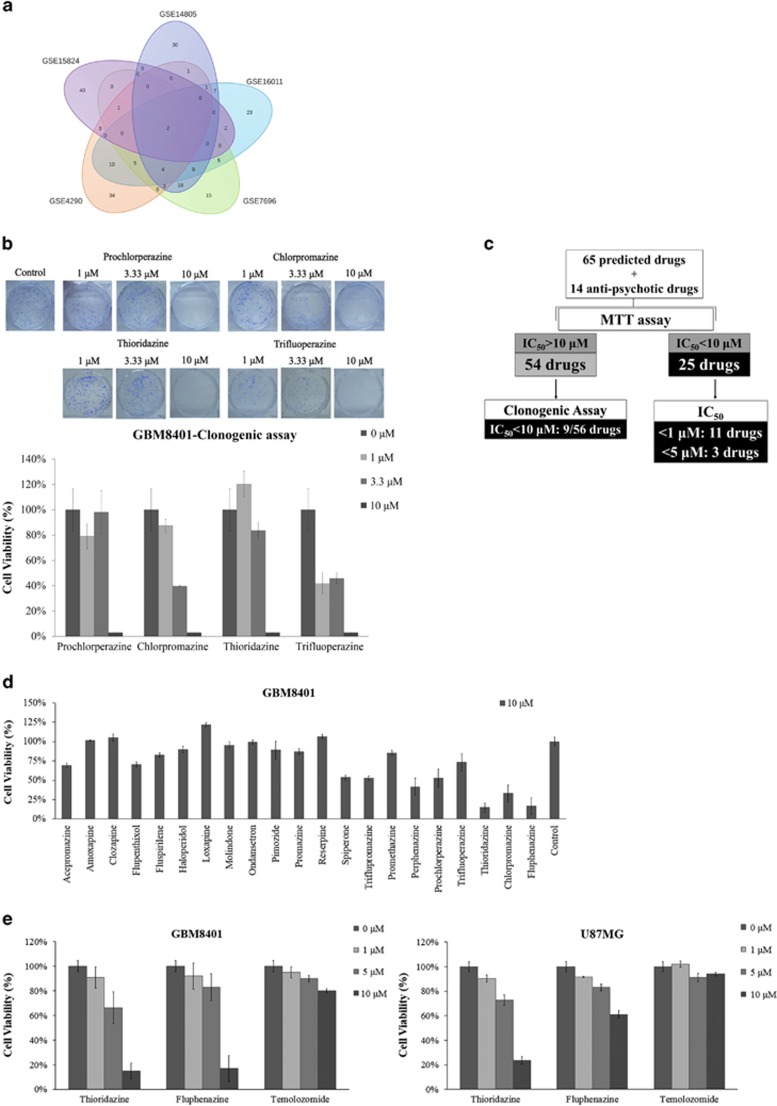
Integrated bioinformatics and biochemical analyses reveal potential drugs for GBM. (**a**) This Venn diagram represents the number of drugs found using the five GBM data sets used to query the Cmap database. (**b**) GBM8401 cells were treated with prochlorperazine, chlorpromazine, thioridazine and trifluoperazine at concentrations ranging from 1 to 10 *μ*M, and cell viability was determined using a clonogenic assay. (**c**) Summary of drugs tested on GBM8401 cells. Of the 215 potential GBM drugs as predicted by Cmap, we randomly selected 65 potential drugs and 14 antipsychotic drugs based on the availability of well-known scientific names and manufacturers, and we tested the cytotoxicity of these drugs in two GBM cell lines via an MTT and/or a clonogenic assay. There were 25 effective drugs (IC_50_<10 *μ*M) in the MTT assay and 9 drugs effective (IC_50_<10 *μ*M) in the clonogenic assay. (**d**) GBM8401 cells were treated with 10 *μ*M various antipsychotic drugs for 72 h. Cell viability was determined by MTT assay. (**e**) GBM8401 and U87MG cells were treated with thioridazine, fluphenazine or temozolomide (the only FDA-approved drug for GBM) at concentrations of 1, 5 or 10 *μ*M for 72 h, respectively. Cell viability was determined via MTT assay

We hypothesized that if a drug treatment could at least partially reverse the gene expression signature of GBM, it might have the potential to inhibit pathways essential in the formation of GBM and could therefore be used to treat GBM. We combined data from five publicly available microarray data sets from the National Center for Biotechnology Information (NCBI) Gene Expression Omnibus (GEO). All five data sets were published previously.^6, 7, 8, 9, 10^ The data sources are summarized in Table 1 and data analysis is described in the Materials and Methods section. Briefly, differentially expressed genes appear in all five data sets, including two upregulated genes (*COL4A1* and *MTHRD2*) and eight downregulated genes (*KIAA1598, CHN2, EPB41L3, GNAI1, GRM3, SH3GL3, RAPGEF5* and *RAB40B*). The total list of differentially expressed genes is found in Supplementary Information, Supplementary Table S4. We then used the GBM gene signatures (determined by comparing the GBM gene expression pattern to the normal brain gene expression pattern) from each data set to query the Cmap (with negative enrichment scores and *P*<0.05) as described in the Materials and Methods section. Figure 1a shows the 215 drugs identified from the fusion of all five data sets, and the list of these drugs is found in Supplementary Information, Supplementary Table S1.

### Characterization of potential drugs for GBM via cell viability and clonogenic assays

To examine whether the 215 predicted drugs have antitumor effects on malignant gliomas, we randomly tested 65 potential drugs in the GBM8401 cells and determined the cell viability by an 3-(4,5-dimethylthiazol-2-yl)-2,5-diphenyltetrazolium bromide (MTT) assay. If the IC_50_ value of the drug was less than 10 *μ*M, we defined the drug as an ‘effective' drug. For example, Supplementary Information, Supplementary Figure S2 illustrates the MTT results from cells treated with thiostrepton, thioguanosine, parthenolide and bepridil; the IC_50_ value for thiostrepton is 0.8 *μ*M. The ‘ineffective' drugs (IC_50_ value >10 *μ*M) were tested further using clonogenic assays. Effectiveness in the clonogenic assay was defined as a reduction in the number of colonies by more than 50% at 10 *μ*M. The quantifications of the clonogenic assays for four antipsychotic drugs, including prochlorperazine, chlorpromazine, thioridazine and trifluoperazine, are illustrated in Figure 1b. We also tested an additional 14 antipsychotic drugs (Figure 1c); in total, 79 drugs were tested. Figure 1c summarizes the results of 34 effective drugs. We classified these effective drugs (25 effective drugs in the MTT assay and 9 effective drugs in the clonogenic assay) into six different functional groups (anti-inflammation, antibiotics, antipsychotics, cardiovascular drugs, chemotherapeutic drugs and others), as summarized in Table 2.

### Analysis of cell viability in GBM cell lines after treatment with antipsychotic drugs

We chose the antipsychotic drugs as our top priority for the following three reasons: (1) antipsychotic drugs can across the blood–brain barrier; (2) one of the studies collected through data mining of the National Health Insurance Research Database in Taiwan noted that patients with schizophrenia have a lower risk of developing cancer.^4^ Thus, we speculated that there might be some correlation between cancer and antipsychotic drugs; and (3) antipsychotic drugs could reverse, at least in part, the gene expression signature of cancer stem-like cells as observed in the Cmap analysis (Supplementary Information, Supplementary Table S5). These arrays of cancer stem-like cells were from three published studies.^11, 12, 13^ Therefore, to systematically examine the cytotoxicity of antipsychotic drugs on malignant glioma, we expanded the collection of the seven antipsychotic drugs predicted by Cmap (acepromazine, amoxapine, chlorpromazine, fluspirilene, prochlorperazine, thioridazine and trifluoperazine) to 21 antipsychotic drugs that had data in the Cmap collections. As shown in Figures 1d and 4 drugs (perphenazine, thioridazine, chlorpromazine and fluphenazine) have an IC_50_ value <10 *μ*M. Because perphenazine and fluphenazine were also effective against GBM cells, we also included these two drugs in Table 2. In particular, thioridazine and fluphenazine resulted in more cytotoxicity. To confirm the effect of the drugs in malignant glioma cell lines, the GBM8401 and U87MG cells were treated with thioridazine and fluphenazine at concentrations ranging from 1 to 10 *μ*M for 72 h, and a dose-dependent decrease in cell growth was observed (Figure 1e). Currently, vascular endothelial growth factor (VEGF) signaling, the epidermal growth factor (EGF) family, the SRC family, platelet-derived growth factor receptorand integrins are being investigated as potential molecular therapeutic targets for GBM treatment.^14, 15, 16^ As increase in phospho-EGFR was observed in fluphenazine-treated GBM8401 cells (Supplementary Information, Supplementary Figure S4), thioridazine instead of fluphenazine was used for the following experiments.

### Effects of thioridazine on cell autophagy in GBM cell lines

To investigate the mechanisms underlying the cytocidal effects of thioridazine, GBM8401 cells were treated with thioridazine at concentrations ranging from 5 to 15 *μ*M or with fluphenazine at concentrations of 10 and 20 *μ*M for 24 h. Following this incubation, we examined protein levels using a microwestern technique, which allows for a quantitative, sensitive and high-throughput (96 different antibodies) assessment of protein levels.^17^ We randomly selected 96 proteins from various pathways. These proteins are primarily involved in the autophagic and cell growth-related pathways (Supplementary Information, Supplementary Table S3). For example, phospho-AMPK (Thr-172) was 3.4-fold upregulated, phospho-mTOR (mammalian target of rapamycin) was 0.58-fold downregulated and phospho-p70S6K (p70 ribosomal protein S6 kinase; Thr-389) was 0.46-fold downregulated. Additional results are summarized in Supplementary Information, Supplementary Table S6. These microwestern results were further validated using the western blot analysis.

**Figure 2:**
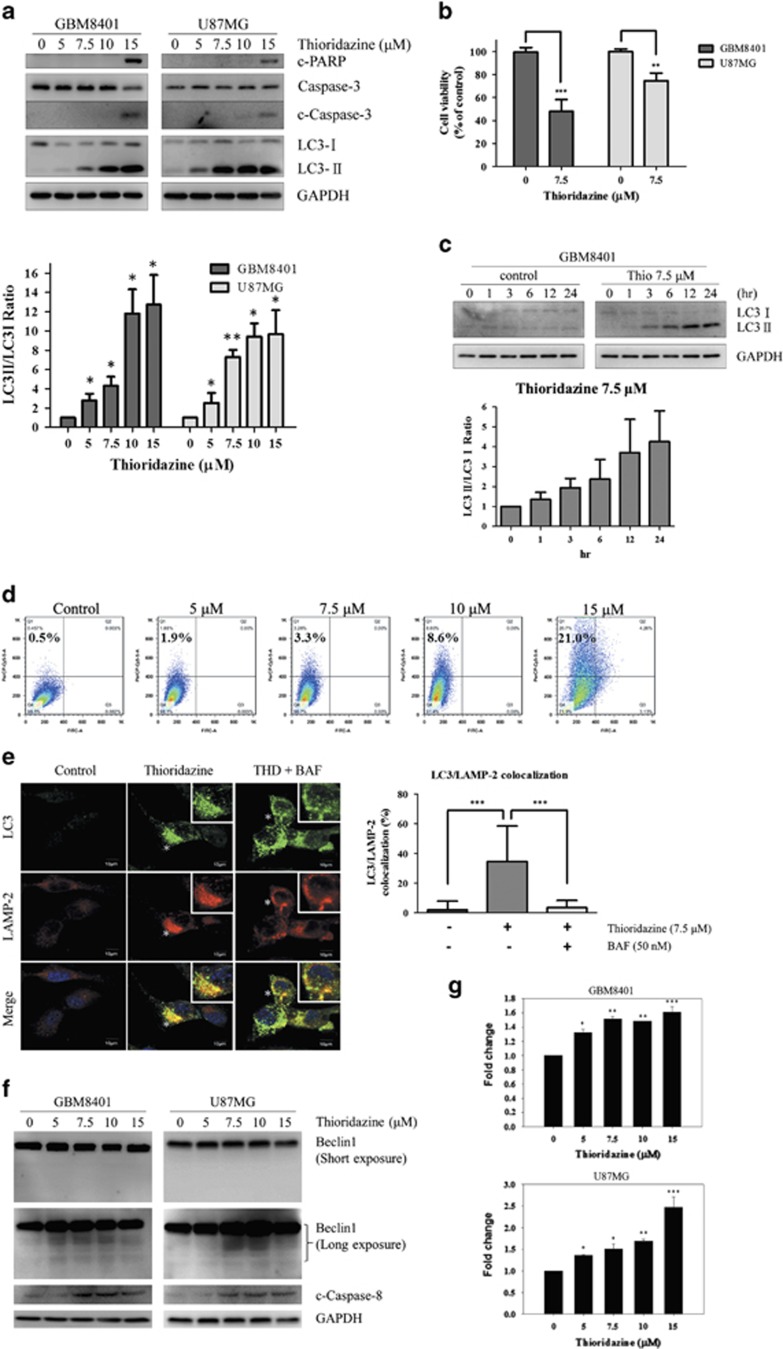
Thioridazine induces autophagy in GBM cells. (**a**) GBM8401 and U87MG cells were treated with thioridazine at 5, 7.5, 10 and 15 *μ*M for 24 h, respectively. c-PARP, Caspase-3, c-Caspase-3, LC3-I and LC3-II were detected by western blotting. GAPDH was used as an internal control. The intensity of LC3-II band density was normalized to LC3I, showing the conversion rate of LC3-I to LC3-II. Bar graph represents the mean of triplicates±S.D. *P<0.05, **P<0.01 compared with the control group. (**b**) GBM8401 and U87MG cells were treated with 7.5 *μ*M thioridazine for 24 h. Bar graph represents the mean of triplicates±S.D. **P<0.01, ***P<0.005 compared with the control group. (**c**) GBM8401 cells were treated with thioridazine at 7.5 *μ*M for 1, 3, 6, 12 and 24 h, respectively. LC3-I and LC3-II were detected by western blotting. GAPDH was used as an internal control. The intensity of LC3-II band density was normalized to LC3I, showing the conversion rate of LC3-I to LC3-II. (**d**) GBM8401 cells were treated with thioridazine at 5, 7.5, 10 and 15 *μ*M for 24 h, respectively. Acridine orange-positive cells were quantified by using flow cytometry. Bar graph represents the mean of triplicates±S.D. **P<0.01 compared with the control group. (**e**) U87MG cells were pretreated with or without 50 nM Bafilomycin A1 for 4 h, and then treated with 7.5 *μ*M thioridazine for 24 h. Cells were stained with antibodies against LC3 (green) and LAMP-2 (red). Asterisk indicates the colocalization of LC3 and LAMP-2 (yellow). Inset shows magnified view of colocalizations. All merged images together with DAPI staining of DNA (blue). Colocalization was analyzed by confocal microscopy ( × 60 magnification). Bar graph represents the mean of duplicates±S.D., as quantified in at least 25 cells for each experimental condition. ***P<0.005. Bars=10 *μ*m. BAF, Bafilomycin A1. (**f**) U87MG and GBM 8401 were treated with thioridazine for 24 h. After treatment, the cleavage of beclin1 and caspase-8 was detected using immunoblotting. The GAPDH was used as an internal control. (**g**) U87MG and GBM 8401 were treated with thioridazine for 24 h. The activity of caspase-8 was determined using luminescence. Bar graph represents the mean of triplicates±S.D. *P<0.05, **P<0.01, ***P<0.005. compared with the control group

It has been shown that GBM cells response better to agents that induce autophagy than apoptosis.^18, 19, 20^ To investigate whether the mechanisms of thioridazine undergo autophagy, apoptosis or both pathways in GBM cells, we used western blot analysis to examine the level of LC3-II (a marker of autophagy), cleaved-Caspase-3 (c-Caspase-3) and cleaved-PARP (c-PARP; markers of apoptosis). The data showed that LC3-II was significantly upregulated in both cell lines treated with thioridazine in a dose-dependent manner, and c-Caspase-3 and c-PARP were also increased at the highest concentration (Figure 2a). It should be noted that thioridazine induces autophagy at 5 and 7.5 *μ*M in a dose-dependent manner, but does not affect apoptosis at these conditions. The data showed that thioridazine has cytotoxic effect at 7.5 *μ*M in GBM cells (Figure 2b). As shown in Figure 2c, thioridazine induced autophagy at 7.5 *μ*M in a time-dependent manner, yet apoptosis was not observed at this condition (data not shown). To verify this observation, flow cytometry was used to quantify acid vesicular organelles (AVOs) after thioridazine-treated GBM cells were stained with acridine orange (AO). It revealed that thioridazine induced AVOs accumulation in a dose-dependent manner (Figure 2d). These results further confirm that thioridazine induces autophagy in GBM cells.

To examine whether thioridazine regulates autophagy flux in GBM cells, immunofluorescence assay was used to analyze the colocalization of LAMP-2 (lysosome-associated membrane protein 2; marker of late endosomes/lysosomes) and LC3 in GBM cells after a 24-h thioridazine treatment. The data showed that thioridazine induced LC3-green punctates (Figure 2e). Moreover, extensive colocalization of LC3-green punctates with LAMP-2 was observed in thioridazine-treated GBM cells. These results support that thioridazine induces autophagy flux in GBM cells. Taken together, these results suggest that autophagy has a critical role in thioridazine-induced cytotoxicity of GBM cells.

It is noteworthy that cleavage of caspase-8 and Beclin1 was observed (Figure 2f) and the activity of caspase-8 was upregulated in GBM8401 and U87MG cells treated with higher concentrations of thioridazine (Figure 2g). AMPK-induced phosphorylation and cleavage of Beclin1 have been reported to cause cleavage of caspase 8, leading to shift autophagy to apoptosis.^21^ These results suggest a possible link between thioridazine-induced autophagy and apoptosis.

### Pathway analysis of thioridazine in GBM cell lines

**Figure 3:**
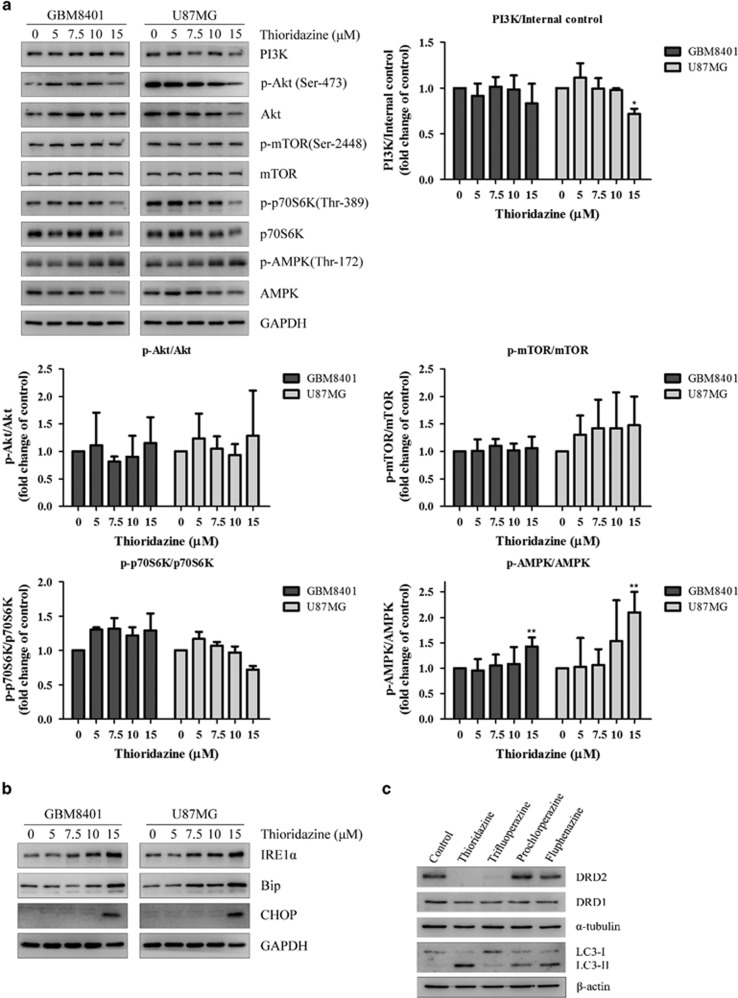
Thioridazine modulates PI3K/Akt/p70S6K signaling pathways and induces ER stress in GBM cells. (**a**) GBM8401 and U87MG cells were treated with thioridazine at 5, 7.5, 10 and 15 *μ*M for 24 h. PI3K, phospho-Akt (Ser-473), phospho-mTOR (Ser-2448) and phospho-S6K (Ser-424) and phospho-AMPK (Thr-172) were detected by western blotting. GAPDH was used as an internal control. The quantification of the western blotting intensity was normalized to internal control intensity using *ImageJ*. Bar graph represents the mean of triplicates±S.D. *P<0.05, **P<0.01 compared with the control group. (**b**) GBM8401 and U87MG cells were treated with thioridazine at 5, 7.5, 10 and 15 *μ*M for 24 h. ER stress markers IRE1*α*, Bip and CHOP were detected by western blotting. GAPDH was used as an internal control. (**c**) GBM8401 cells were treated with different antipsychotic drugs at 10 *μ*M for 24 h. DRD2, DRD1, LC3-I and LC3-II were detected by western blotting. *α*-tubulin and *β*-actin were used as internal controls

We examined the phosphatidylinositol 3-kinase (PI3K)/Akt/mTOR pathway, which has known roles in the regulation of autophagy. Interestingly, the data showed that thioridazine affected the PI3K/Akt pathway at 15 *μ*M and PI3K, phospho-Akt (Ser-473) and phospho-p70S6K (Ser-424) were downregulated. However, phospho-AMPK (Thr-172) was upregulated in a dose-dependent manner (Figure 3a). These results suggest that the major pathway of thioridazine-induced autophagy may be through AMPK activation in GBM cells. The microwestern (Supplementary Information, Supplementary Table S6) and western (Supplementary Information, Supplementary Figure S3) results indicate that phospho-regulatory associated protein of MTOR (Raptor) was downregulated. Recently, phosphorylation of Raptor, a component of the mTOR complex 1, by AMPK has been reported.^22^ Although the expression of p-mTOR was not significantly different (Figure 3a), thioridazine-induced autophagy may be also through AMPK-induced suppression of Raptor. It is noteworthy that total AMPK was downregulated, although phospho-AMPK (Thr-172) was upregulated (Figure 3a; Supplementary Information, Supplementary Table S6). Recently, it has been reported that thioridazine decreased protein expression of translationally controlled tumor protein (TCTP),^23^ and depletion of TCTP led to phosphorylation of AMPK at Thr-172.^24^ Mmi1, the yeast homolog of mammalian TCTP, acted as a proteasome inhibitor, protecting protein substrates from proteasomal degradation.^25^ Another possibility may be due to the regulation of AMPK by the ubiquitin proteasome system, in which activating AMPK using AMPK agonists increases expression of ubiquitin ligases (MuRF1 and MAFBx/Atrogin-1), and enhances ubiquitination of AMPK.^26^

Recent report demonstrates that ER stress might be an upstream link for autophagy- and apoptosis-mediated cell death in gliomas.^27^ As shown in Figure 3b, unfolded protein response activation (accumulation of inositol-requiring enzyme 1 alpha (IRE1*α*)) and accumulation of ER stress-associated proteins (binding immunoglobulin protein (Bip) and C/EBP homologous protein (CHOP)) were observed in thioridazine-treated GBM cells. These results suggest that thioridazine induces ER stress and autophagy.

### Effects of thioridazine on DRD2 activity in GBM cell lines

Thioridazine is known to target dopamine receptors in patients with schizophrenia and psychosis.^28, 29^ We sought to investigate whether different phenothiazine derivatives (thioridazine, trifluoperazine, prochlorperazine and fluphenazine) might affect dopamine receptor D2 (DRD2) expression in GBM cells. The data showed that both thioridazine and trifluoperazine downregulated the expression of DRD2 in GBM cells, whereas prochlorperazine and fluphenazine had no effect on DRD2. These results suggest that different phenothiazine derivates have different effects on DRD2 in GBM cells. We further identified whether DRD2 expression was associated with autophagy. The data showed that LC3-II was upregulated in GBM cells treated with thioridazine, prochlorperazine and fluphenazine, but not trifluoperazine (Figure 3c). These results suggest that the autophagy pathway induced by these antipsychotic drugs is independent of DRD2 expression in GBM.

### Analysis of cell viability in GBM cancer stem-like cells after treatment with anti-psychotic drugs

**Figure 4:**
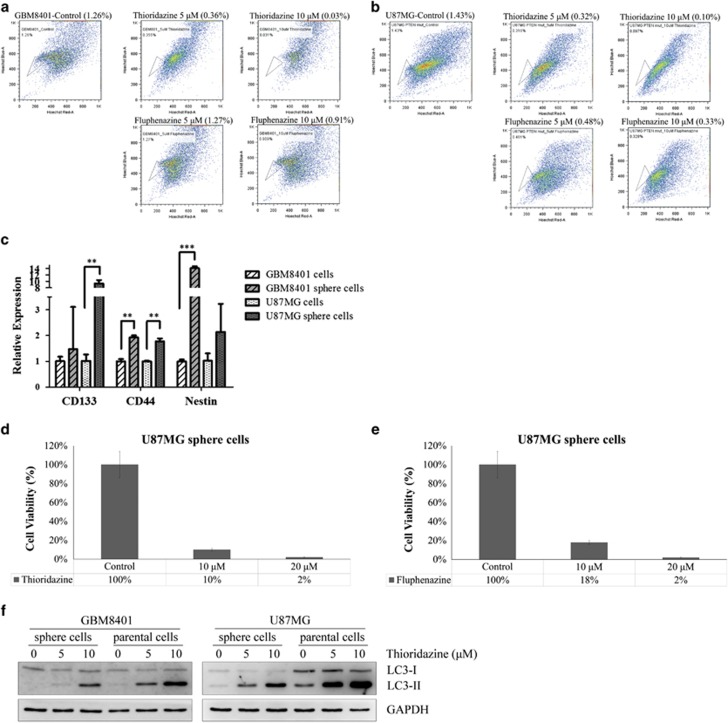
Thioridazine and fluphenazine reduce the percentage of cancer stem-like cell side population and affect the viability of U87MG sphere cells. (**a** and **b**) The cancer stem-like cells side population was significantly decreased by treatment with thioridazine (10 *μ*M) from 1.26 to 0.03% in GBM8401 cells and from 1.43 to 0.1% in U87MG cells, as determined by side population assay. (**c**) Quantification of GSCs gene expression with GBM8401 and U87MG sphere cells by TaqMan real-time qRT-PCR, including CD133, CD44 and Nestin. Bar graph represents the mean of triplicates±S.D. **P<0.01, ***P<0.005. U87MG sphere cells were treated with 10 and 20 *μ*M thioridazine (**d**) and fluphenazine (**e**) for 24 h, respectively. The viability of the U87MG sphere cells was determined by counting cells after trypan blue staining. (**f**) Both sphere cells and parental cells of GBM8401 and U87MG were treated with thioridazine at 5 and 10 *μ*M for 24 h, respectively. LC3-I and LC3-II were detected by western blotting. GAPDH was used as an internal control

Our bioinformatics analysis indicated that anti-psychotic drug signatures have a negative (or reverse) correlation with cancer stem-like cell signatures^11, 12, 13^ as shown in Supplementary Information,Supplementary Table S5. We first used a flow cytometry-based side population technique to isolate the cancer stem-like cells from the GBM8401 and U87MG cells to examine the potential anti-cancer stem-like cells effects in response to these anti-psychotics drugs. Treatment with thioridazine reduced the percentages (> 50%) of the side population cells in both the GBM8401 and U87MG cells. However, fluphenazine only reduced the percentage of the side population cells in the U87MG cells but not in the GBM8401 cells (Figures 4a and b). In short, we observed that thioridazine was more effective than fluphenazine against the GBM stem-like cells based on the side population assay. To examine whether GBM sphere cells have cancer stem cell properties, TaqMan real-time PCR was used to detect the gene expression of glioblastoma stem cell (GSC) markers in GBM sphere cells. The expression of the GSCs markers, including CD133, CD44 and Nestin, were enhanced in GBM sphere cells (Figure 4c). These results indicate that GBM sphere cells have cancer stem cell properties.

To investigate whether thioridazine or fluphenazine possess anticancer stem-like cell properties, U87MG cells were cultured in HEScGRO serum-free medium for the duration of the tumor spheroid assay. Sphere cells (cancer stem-like cells) were treated with thioridazine (Figure 4d) or fluphenazine (Figure 4e) at 10 and 20 *μ*M for 24 h. The cell viability was significantly reduced. As described previously, we found that thioridazine induces autophagy in GBM cells; thus, we further investigated whether thioridazine induces autophagy in GBM sphere cells. After a 24-h treatment with thioridazine, LC3-II was significantly upregulated in GBM sphere cells in a dose-dependent manner (Figure 4f). These results suggest that thioridazine induces autophagy in GBM sphere cells.

### *In vivo* examination of anti-GBM effect of thioridazine

**Figure 5:**
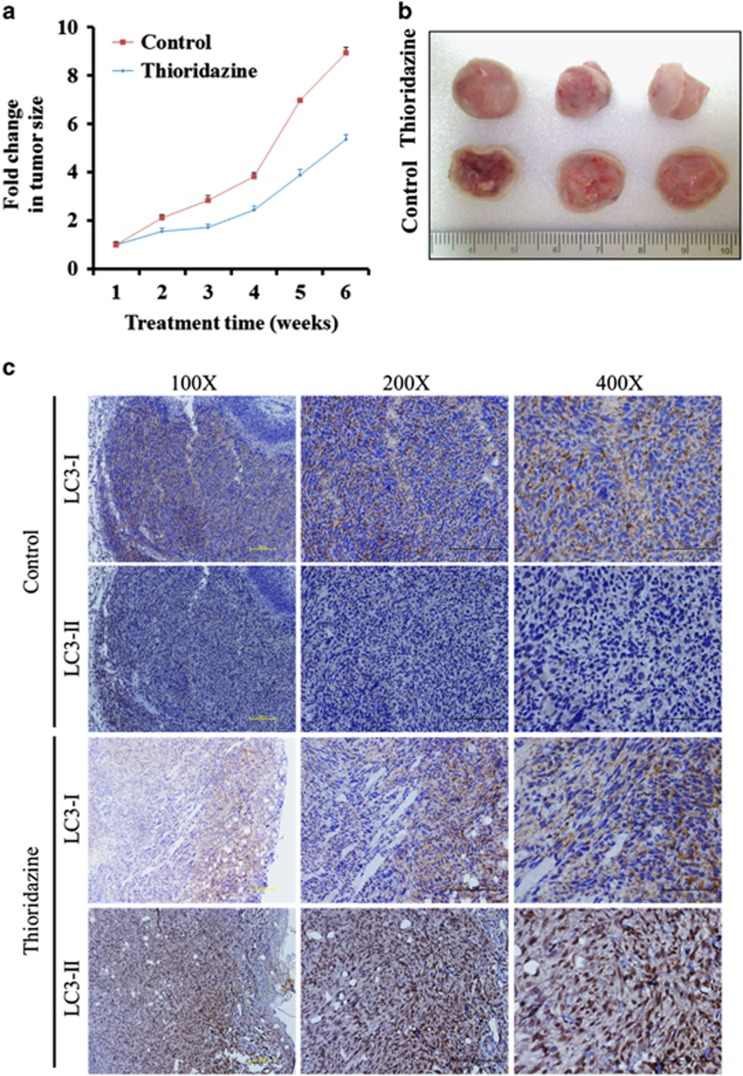
Thioridazine suppresses GBM tumorigenesis and induces autophagy *in vivo*. (**a** and **b**) U87MG cells (1 × 10^6^ cells/injection) were subcutaneously implanted into NOD/SCID mice and subdivided into two groups: control (DMSO) and thioridazine (5 mg/kg/day, 5 days/week). Mice were treated by intraperitoneal injections. The tumor size was measured using caliper on a weekly basis. Mice treated with thioridazine exhibited a significantly lower tumor burden as compared with those in the control group (*n*=3 per treatment group). (**c**) Representative photographs of tumor biopsies obtained from control and thioridazine-treated animals. LC3-I and LC3-II were detected by immunohistochemistry

To detect the effect of thioridazine *in vivo*, we tested tumor growth in xenograft mice model. As shown in Figures 5a and b, the tumor size was significantly smaller than those in the control group. To further study the relationship of autophagy in GBM, tumor sections from different groups of mice were examined. A very high expression of LC3-II was observed in thioridazine-treated mice as compared with control mice using immunohistochemistry (Figure 5c). Overall, we provide strong evidence of thioridazine with autophagy in anti-GBM effect.

## Discussion

Using the Cmap, a powerful bioinformatics tool in drug discovery, we showed that thioridazine, an antipsychotic drug used to treat schizophrenia and psychosis, is an anti-GBM agent. Treatment with thioridazine decreases the viability of GBM cells and cancer stem-like cells. In addition, thioridazine induces autophagy and induces apoptosis at the high concentration. These phenomena may function through G protein-coupled receptors.

TMZ methylates DNA at the N-7 or O-6 positions of guanine residues, damaging the DNA and leading to death in tumor cells. The methylation of the O^6^-methylguanine-DNA methyltransferase (MGMT) gene promoter has been used to predict tumor response to TMZ.^30^ MGMT is a DNA-repair protein that prevents the chemotherapy-induced DNA damage by maintaining the structural integrity of an O^6^-alkylated base. However, about half of all GBM patients obtained an unmethylated MGMT promoter and therefore respond poorly to TMZ.^14, 30^

In this study, we set up three bioinformatics analyses to guide our drug-repurposing pipeline. First, many genomic approaches were used to annotate the disease-causing genes in GBM.^31, 32^ Here, we analyzed the GBM gene signature from five microarray studies^6, 7, 8, 9, 10^ and used the selected signatures to query the Cmap database. We did not focus on those intersection drugs (Supplementary Information, Supplementary Table S1), for example, 1,4-chrysenequinone and 0173570-0000, because these drugs have not been tested on human subjects. These compounds may take a long time to develop into usable drugs for the treatment of GBM. Second, cancer stem cells are thought to have a role in GBM resistance and recurrence.^33^ Cmap analysis predicted thioridazine to inhibit these GBM cancer stem-like cells (Supplementary Information, Supplementary Table S5). Finally, we took advantage of the multitude of literature available and mined data from the National Health Insurance Research Database in Taiwan and set up our second criteria for drug prioritization. In this study, we noticed that patients with schizophrenia (~59 000) have a lower risk of developing cancer than the control patients (~178 000).^4^ On the basis of these data integrations and biochemical analyses, we subsequently demonstrated that thioridazine was effective in decreasing the growth of GBM cells and GBM stem-like cells. Moreover, a study reported that thioridazine selectively targets human somatic cancer stem cells capable of leukemic disease initiation, whereas it had no effect on normal blood stem cells.^34^ This further supports our hypothesis that thioridazine is an anti-GBM agent that targets GBM cancer stem-like cells.

Thioridazine has been shown to induce DNA fragmentation and increase Caspase-3 activity; however, higher doses (6~50 *μ*M) are needed to elicit these results in glioma cells.^35^ Here, we clearly demonstrate that thioridazine induces both autophagy and apoptosis in GBM cells. Our data, however, point that autophagy might be a major mechanism underlying the effects of thioridazine. As in GBM sphere cells, treatment of thioridazine also induces autophagy, but not apoptosis. Further, autophagy occurred earlier than apoptosis in GBM cells. In addition, thioridazine appears to inhibit GBM tumor growth effectively and induces autophagy *in vivo*, suggesting that thioridazine induced autophagy is cytocidal role in GBM.

Using Cmap as an alternative tool for drug discovery could lead to FDA approval of old drugs to treat other disorders. In addition, 443 drugs, which include thioridazine, in the Cmap database have been tested in humans according to the National Health Insurance Research Database in Taiwan. This supports our drug discovery pipeline in drug repurposing to quickly survey survival data and side effect of patients before conducting clinical trials looking for new indications.

## Conclusions

We not only repurposed the antipsychotic drug thioridazine as a potent anti-GBM and anti-GBM cancer stem-like cells agent, but also provided a new strategy to search for drugs with anticancer and anticancer stem-like cell properties.

## Materials and Methods

### Cell lines and cell culture

Dulbecco's modified Eagle's medium (DMEM, Life Technologies, Grand Island, NY, USA; 12100-046) supplemented with 10% fetal bovine serum (Life Technologies, 10099-133), 2 mM L-glutamine (Life Technologies, 25030-081), 100 U/ml penicillin (Life Technologies, 15140-163) and 100 *μ*g/ml streptomycin (Life Technologies, 15140-163) were used to maintain human glioblastoma GBM8401 and U87MG cells (Bioresource Collection and Research Centre, BCRC, Taiwan, 60163, 60360, respectively) in a humidified incubator at 37 °C with a atmosphere of 5% CO_2_. The cells were regularly subcultured every 2–3 days.

### Chemicals

Acepromazine (A7111), Amoxapine (A129), Clozapine (C6305), Flupenthixol (F114), Fluspirilene (F100), Haloperidol (H1512), Loxapine (L106), Molindone (M1818), Ondansetron (O3639), Pimozide (P1793), Reserpine (R0875), Spiperone (S7395), Triflupromazine (T2896), Prochlorperazine (P9178), Trifluoperazine (T8516), Thioridazine (T9025), TMZ (T2577), Chlorpromazine (C8138) and Fluphenazine (F4765) were purchased from Sigma. Perphenazine (125), Thiostrepton (522), Thioguanosine (347), Parthenolide (550) and Bepridil (368) were purchased from Prestwick. Promazine (46674) and Promethazine (46682) were purchased from Fluka.

### Collection and analysis of GBM microarray data sets

Five cohorts of GBM patients (GSE4290, GSE7696, GSE14805, GSE15824 and GSE16011)^6, 7, 8, 9, 10^ were used in this study; in total, 397 microarrays (Table 1) were obtained from the NCBI GEO website (http://www.ncbi.nlm.nih.gov/geo/). Among these five data sets, the GSE14805 data set was subjected to analysis using the Affymetrix HT-HG-U133A chip, and the other data sets were analyzed using the Affymetrix HC-U133 plus 2.0 chip (Table 1). For example, the GSE4290 data set was obtained from Fine *et al.;*^6^ this data set included 81 GBM samples and 23 samples from epilepsy patients, as nontumor controls. This data set was generated using Affymetrix HC-U133 plus 2.0 chips, which include 54 675 probe sets. However, the samples used in the Cmap were profiled using Affymetrix HG-U133A gene chips, which include 22 283 probe sets. Therefore, Cmap analysis was used to filter out the probe sets that do not belong to the HG-U133A platform. To analyze the GBM microarrays, the raw fluorescence intensity data within the raw CEL files of these data sets were preprocessed and normalized using the Bioconductor Affy package, based on the Robust Multichip Average algorithm with R language from Bioconductor (http://www.bioconductor.org). The logarithm to the base 2 of the intensity in the tumor and the control sample was used as the expression value for each probe.

### *In silico* drug screening via the Cmap

To identify significant differentially expressed genes from microarrays with GBM and normal brain samples, we used the two sample *t*-test (*P*-value <0.0001) with at least fourfold change cutoff. For each GBM sample and nontumor sample, 22 283 means of expression (one for each probe set) were calculated. The probe sets with significant mean ratios (greater than a fourfold change) were selected as the genes that were differentially expressed. The details of the significant probe sets are summarized in Table 1. These probe sets were used to query up to 7000 expression profiles stored in the Cmap database. For example, to identify the differentially expressed probe sets from the GSE4290 data set using the volcano plot method, 120 upregulated and 286 downregulated probe sets were selected and used to query the Cmap. To identify potential GBM treatments, only drugs with a *P*-value of less than 0.05 and a negative enrichment score were retained. The details of the potential drugs from the five GBM data sets are shown in Supplementary Information, Supplementary Table S1.

### Cell viability assay

The viability of cell was measured using the MTT assay. Briefly, cells (1500 cells/well) in 96-well plates were treated with various concentrations of the tested agents for various time points. After treatment, 10% stock MTT reagent (0.02 mg MTT from Sigma in 10 ml phosphate-buffered saline (PBS) for stock reagent, M5655) was added to each well. After another 3–5 h, the cells were dissolved in DMSO for 10 min at room temperature. The absorbance of each well at 570 nm was measured using a microplate reader.

### Clonogenic assay

GBM8401 cells were seeded at a density of 1 × 10^3^ cells per well in six-well plates. Each well was given 2 ml DMEM medium to culture the GBM8401 cells. After 24 h, thioridazine, fluphenazine and other drugs were added, and cells were treated for 14 days. The culture medium was changed on days 4, 8 and 12, and fresh drugs were added at this time. After treatment, cells were washed carefully with PBS. The colonies were then incubated with fix solution (acetic acid:methanol=1 : 3). After being stained with 0.5% crystal violet in methanol, the cells were washed with tap water. The colonies were counted manually.

### Microarray analysis

Human GBM cell line, GBM8401 cells, were treated with 10 *μ*M thioridazine for 6 h. After treatments, total RNA was isolated using the RNeasy Mini kit (QIAGEN, Valencia, CA, USA; 74106). Gene expression was analyzed using the Affymetrix U133plus 2 array. The quality of the total RNA for microarray analysis was determined using UV spectrophotometer and Agilent 2100 Bioanalyzer and had an OD260/OD280 ratio ranging from 1.9 to 2.1. Total RNA was used to synthesize cDNA and was then converted to cRNA. cRNA was labeled, fragmented and quality-checked using Bioanalyzer. According to instructions provided by Affymetrix (http://www.affymetrix.com/support/technical/manuals.affx), hybridization, washing and staining procedures were performed. The images transformed into the text files contain the intensity information using the GeneChip Operating Software, developed by Affymetrix. The microarray data sets were determined using the GeneSpring 7.31 software (QIAGEN, Agilent, Santa Clara, CA, USA).

### Consensus PathDB data analysis

To analyze the thioridazine-elicited effect on GBM8401 cells, we first selected the differentially expressed genes, which were defined as fold changes greater than 1.75 with the greatest difference in expression levels, from the Affymetrix HG-U133_Plus_2 microarray profiling, and these genes were then subjected to ConsensusPathDB (http://cpdb.molgen.mpg.de/) analysis (Supplementary Information, Supplementary Table S2). The second set of differentially expressed genes subjected to the ConsensusPathDB analysis was obtained from the original Cmap data sets, which included 14 thioridazine-treated Affymetrix U133A arrays. Genes were selected based on at least a twofold change and appearance in at least 3 of the 14 arrays.

### Microwestern

GBM8401 cells were treated with thioridazine at concentrations ranging from 5 to 15 *μ*M, or fluphenazine 10 and 20 *μ*M for 24 h, respectively. At the end of treatment, cells were lysed with lysis buffer (240 mM Tris-acetate, 1% sodium dodecyl sulfate (SDS), 0.5% glycerol and 5 mM EDTA), and protein expression was examined via microwestern (Supplementary Information, Supplementary Figure S1) as previously described.^17^ The position of primary antibodies is summarized in Supplementary Information, Supplementary Table S3. Primary antibodies, including stress-activated kinases (SAPK)/c-Jun N-terminal kinases (JNK; 9258), Src (2109), bcl-2-associated X protein (2772), c-Myc (5605), c-Raf (9422), glycogen synthase kinase 3 beta (9315), heat shock protein 90 (4874), janus kinase 2 (3230), p38*α* (2371), p70S6K (2708), phospho-AMPK (Thr-172; 4188), phospho-p38 (Thr-180/Tyr-182; 4511S), phospho-PDGF*β* (Tyr-1009) (3124S), phospho-Raptor (Ser-792; 2083), phospho- retinoblastoma protein (Ser-780; 3590S), phospho-SAPK/JNK (Thr-183/Tyr-185; 4668S), phospho-Tuberin/ tuberous sclerosis 2 (Thr-1462; 3617) and phospho-VEGF Receptor 2 (Tyr-1059; 3817S), were obtained from Cell Signaling (Danvers, MA, USA). All other antibodies were obtained from Millipore (Bedford, MA, USA).

### Western blotting analysis

Cells were lysed with lysis buffer (Thermo, Waltham, MA, USA; 89900) containing 25 mM Tris-HCl (pH 7.6), 150 mM NaCl, 1% NP-40, 1% sodium deoxycholate and 0.1% SDS. Total protein was isolated and subjected to SDS-polyacrylamide gel electrophoresis and electrotransferred on polyvinylidene difluoride membranes (Millipore, IPVH00010). The primary antibodies, including Bip (1 : 1000; 3177), CHOP (1 : 1000; 2895), IRE1*α* (1 : 1000; 3294), phospho-mTOR (Ser-2448; 1 : 1000; 5536S), mTOR (1 : 1000; 2983S), PI3K (p110*α*; 1 : 1000; 4249S), phospho-Akt (Ser-473; 1 : 1000; 4060S), Akt (1 : 1000; 2938S), phospho-p70S6K (Ser-424; 1 : 1000; 9205S), p70S6K (1 : 1000; 2708S), phospho-AMPK (Thr-172; 1 : 1000; 2535S), AMPK (1 : 1000; 2532S), c-PARP (1 : 1000; 5625P), c-Caspase-3 (1 : 1000; 9664S) and Caspase-3 (1 : 1000; 9662S), p-Raptor (Ser-792; 1 : 1000; 2083S), Beclin1 (1 : 1000; 3495S) and c-Caspase-8 (1 : 1000; 8592) were obtained from Cell Signaling. DRD2 (1 : 1000; ab88074) and Dopamine receptor D1 (DRD1, 1 : 1000; ab40653) and *β*-actin (1 : 1000; ab3280) were from Abcam (Cambridge Science Park, UK), and LC3 was from Abgent (San Diego, CA, USA; 1 : 1000; AP1802a), and GAPDH (1 : 10 000; GTX100283) and *α*-tubulin (1 : 5,000; GTX112141) were from GeneTex (Irvine, CA, USA). The secondary antibodies for anti-rabbit and anti-mouse horseradish preoxidase (HRP) conjugation were from Chemicon (Shinagawa-ku, Tokyo, Japan; 12–348 and 12–349, respectively). The protein detection was performed with enhanced chemiluminescence (GE Healthcare, Pittsburgh, PA, USA; RPN2108) method captured by a Luminescence Imaging System (LAS-4000, Fuji Photo Film Co, Ltd, Minato-ku, Tokyo, Japan). After scanning western blots into a computer, individual bands were analyzed for optical density using *ImageJ*.

### Determination of caspase 8 activity

U87MG and GBM8401 cells seeded at a density of 2.5 × 10^4^ cells per well in 96-well plates were treated with 5–15 *μ*M thioridazine. After 24 h, the substrate of caspase 8 was added to the samples and incubated for 30 min. The activity was detected using luminescence.

### Detection of AVOs with AO staining using flow cytometry

AO is a fluorescent weak base that accumulates in acidic spaces and fluoresces bright red.^36^ AVOs, representing formation of autophagosomes and autolysosomes, were determined using flow cytometry after cells were stained with AO. After drug treatments, cells were stained with AO (1 *μ*g/ml, Sigma, St. Louis, MO, USA; A6014) for 20 min at room temperature, washed twice with PBS, removed from the dish with trypsin-EDTA (Life Technologies, R-001-100) and collected in phenol red-free growth medium. AVOs were measured using a BD FACSCanto, followed by analysis with the FACS Diva software (BD Bioscience, San Jose, CA, USA).

### Immunofluorescence staining

U87MG cells on the coverslips in 12-well plate were pretreated in the presence and absence of 50 nM Bafilomycin A1 (Sigma, B1793) for 4 h before 7.5 *μ*M thioridazine treatment for 24 h. After being fixed with 3.7% formaldehyde in Hank's balanced salt solution (HBSS) at room temperature, the cells were blocked with 5% bovine serum albumin (Millipore, 810037) in HBSS. Followed by incubated with anti-LC3 antibody (rabbit polyclonal IgG; 1 : 100; Abgent, AP1802a) and LAMP-2 (mouse monoclonal IgG; 1 : 100; Abcam, ab25631), the primary antibodies were examined using Alexa Fluor 488-conjugated anti-rabbit antibody (1 : 1000; Life Technologies, A11008) and Alexa Fluor 568-conjugated anti-mouse antibody (1 : 1000; Life Technologies, A11004). DAPI (2 *μ*g/ml, Sigma, D9542) was used to stain the nuclei after being mounted with ProLong Gold antifade reagents (Life Technologies, P36930). The images were obtained using an Olympus BX61 confocal microscope using × 60 objective.

### Identification of side population by using flow cytometry

U87MG and GBM8401 cells were treated with thioridazine at 5 and 10 *μ*M or fluphenazine at 5 and 10 *μ*M for 24 h, respectively. Cells were detached by trypsinization (Life Technologies, 12604013), following by resuspension at 1 × 10^6^ cells/ml in 3% fetal calf serum and 10 mM HEPES-supplemented HBSS (Life Technologies, 15630-080). Cells were then incubated with Hoechst 33342 (Sigma, 14533) at 37 °C for 90 min, alone or with 50 *μ*M verapamil (Sigma, V4629), for inhibition of the verapamil-responsive ABC transporter. Cells were then centrifuged at 300 × *g* and resuspended in ice-cold HBSS. Cells were placed on ice to inhibit efflux of the Hoechst dye, followed by addition of 1 *μ*g/ml propidium iodide (BD Bioscience, 556463) to distinguish dead cells. Finally, a single-cell suspension was generated by filtering the cells through a 40-*μ*m cell strainer (BD Bioscience) to obtain. Dual-wavelength analysis and purification were then performed using a dual-laser FACS Vantage SE machine (BD Bioscience). We excited Hoechst 33342 with a 355-nm UV light, followed by emission of blue fluorescence with a 450/20 band-pass filter as well as red fluorescence with a 675-nm edge filter long-pass. A 610-nm beam-splitter or so-called ‘dichroic mirror shortpass' was used to separate the emitted light per wavelength.

### Real-time quantitative RT-PCR

Total RNA was extracted from U87MG cells and sphere cells using the NucleoSpin RNA II kit (MACHEREY-NAGEL, Bethlehem, PA, USA) according to the manufacturer's protocol. For cDNA synthesis, RNA was reverse-transcribed into cDNA using ThermoScript RT-PCR System (Invitrogen, Grand Island, NY, USA). Gene expression was quantified by real-time quantitative RT-PCR using TaqMan probe (Life Technologies). The relative quantities of gene mRNA against an internal control, GAPDH, were detected by following a Δ*C*_t_ method. The difference (Δ*C*_t_) between the mean values in the duplicated samples of target gene and those of GAPDH were calculated by Microsoft Excel and the relative quantified value was expressed as 2^−Δ*C*t^.

### Tumor spheroid assay

To evaluate the formation of tumor spheroids, we cultured GBM8401 and U87MG cells in HEScGRO serum-free medium (Chemicon SCM020) supplemented with NeuroCult NS-A (STEMCELL Technologies, Vancouver, BC, Canada; 5751), 20 ng/ml human epidermal growth factor and 10 ng/ml hFGF. Cells were seeded at 1 × 10^3^ cells/ml in 12-well, low-adhesion plates. The generated spheroids (tight, spherical, non-adherent cell-masses >90 *μ*m in diameter) were counted, followed by measurement of at least 50 spheroids per group using an ocular micrometer. For the secondary spheroid formation assay, we mechanically dissociated the primary spheroids and processed exactly as for the primary assay. To estimate the percentage of spheroid-forming cells, we seeded one cell per well in 96-well plates.

### Xenograft experiments

U87MG cells (1 × 10^6^ cells/injection) were subcutaneously implanted into the flank of NOD/SCID mice. The mice were randomly distributed into two groups (three mice/group): control (DMSO as the vehicle) and thioridazine (5 mg/kg/day, 5 days/week). The animal study was performed under the strict adherence to the Labortory Animal Use Protocol by Taipei Medical Univeristy (Protocol Approval number: LAC-2013-0086). The treatment began 1 week post-tumor implantation. The tumor volume was then measured using caliper on a weekly basis. Tumor volume was measured using a conventional formula: volume (*V*)=(*W*^2^ × *L*)/2 where *W*=width and *L*=length. The changes in tumor volume (fold change) was then plotted against time. Representative photographs of tumor biopsies obtained from control and thioridazine-treated animals.

### Histology and immunohistochemical staining

Tumor tissues were collected from xenografted mice, and then fixed with formalin and embedded in paraffin. After antigen retrieval of deparaffinized slides, they were probed with anti-LC3-I antibody (1 : 100; Cell Signaling, 4599P) or anti-LC3-II antibody (1 : 100; Cell Signaling, 3868P). Later slides were washed and incubated in a biotinylated link universal antiserum, and then in HRP-conjugated streptavidin (LSAB one kit, GeneCopoeia, Rockville, MD, USA; VB-6015). Slides were rinsed and color-developed with the chromogen, 3, 3-diaminobenzidine hydrochlorides. Lastly, tissue sections were washed with distilled water, counterstained with Mayer's hematoxylin and mounted with DPX mountant. Pictures were then taken with a Photometrics CoolSnap CF color camera (Nikon, Lewisville, TX, USA).

## Figures and Tables

**Table 1:** Summary of five cohorts of GBM data sets used for Cmap analysis

**GEO no.**	**GSE4290**	**GSE7696**	**GSE14805**	**GSE15824**	**GSE16011**
Pubmed ID	16616334	18565887	19139420	21406405	19920198
Journal	Cancer Cell	Clin Oncol	Neuro Oncol	Cancer Res	Cancer Res
Up probe sets no.	120	76	364	67	462
Down probe sets no.	286	194	506	44	538
Brain tumor (GBM)	81	70	34	12	159
Normal brain	23	4	4	2	8
Array platform	HG-U133_Plus_2	HG-U133_Plus_2	HT_HG-U133A	HG-U133_Plus_2	HG-U133_Plus_2

The microarray data were downloaded from the GEO. The differentially expressed gene signatures from each data set were used to query the Cmap

**Table 2:** Thirty-four effective drugs were classified into six different functional groups

**Function**	**Drug name**	**PubMed search**		**Experiment**	
		**Brain cancer**	**Other cancers**	**MTT IC**_**50**_ **(*μ*M)**	**Clonogenic IC**_**50**_ **(*μ*M)**
Anti-inflammation	Tanespimycin	V	V	<0.1	ND
	15-delta prostaglandin J2	V	V	>10	<10
	Luteolin	V	V	<10	<10
	Parthenolide	V	V	5~10	<10
Antibiotics	Thiostrepton	V	V	<1	ND
	Antimycin A	V	V	<1	<10
	Sulconazole	N/A	N/A	>10	<10
	Cycloserine	V	V	<10	ND
Antipsychotics	Chlorpromazine	V	V	5~10	<3.3
	Trifluoperazine	V	V	>10	<1
	Thioridazine	V	V	5~10	<10
	Prochlorperazine	V	V	>10	<10
	Fluphenazine	V	V	5~10	ND
	Perphenazine	V	V	5~10	<10
Cardiovascular drugs	Piperlongumine	V	V	<1	ND
	Propafenone	N/A	V	>10	<10
	Phenoxybenzamine	V	V	>10	<10
	Amiodarone	V	V	>5	ND
Chemotherapeutic drugs	Daunorubicin	V	V	<1	ND
	Camptothecin	V	V	<0.1	<10
	Thioguanosine	V	V	<5	ND
	8-azaguanine	V	V	<10	ND
	GW-8510	N/A	V	<3	ND
	Ellipticine	V	V	<1	ND
Others	Cloperastine	N/A	N/A	>10	<10
	Nitrarine dihydrochloride	N/A	N/A	>10	<10
	Emetine	V	V	<0.1	ND
	Tyloxapol	N/A	V	5~10	ND
	Norcyclobenzaprine	N/A	N/A	>10	<10
	Trichostatin A	V	V	<0.33	ND
	Pyrvinium	N/A	V	<0.1	ND
	Bepridil	V	V	5~10	ND
	Verteporfin	N/A	V	<3.3	ND
	Vorinostat	V	V	<1	ND

Abbreviations: ‘N/A', no evidence linked to cancer in PubMed; ‘ND', not determined

## Abbreviations

AMPK: AMP-activated protein kinase
AVOs: acidic vesicular organelles
BCRC: Bioresource Collection and Research Centre
Bip: binding immunoglobulin protein
c-Caspase-3: cleaved-Caspase-3
CHOP: C/EBP homologous protein
Cmap: connectivity map
c-PARP: cleaved-Poly (ADP-ribose) polymerase
DAPI: 4',6-diamidino-2-phenylindole
DMEM: Dulbecco's modified Eagle's medium
DMSO: dimethyl sulfoxide
DRD1: dopamine receptor D1
DRD2: dopamine receptor D2
EGF: epidermal growth factor
GBM: glioblastoma
GEO: Gene Expression Omnibus
GFP: green fluorescent protein
GSCs: glioblastoma stem cells
HBSS: Hank's balanced salt solution
HRP: horseradish peroxidase
IRE1*α*: inositol-requiring enzyme 1 alpha
JNK: c-Jun N-terminal kinases
LAMP-2: lysosome-associated membrane protein 2
LC3: microtubule-associated protein 1 light chain 3 (MAP1LC3)
MGMT: O^6^-methylguanine-DNA methyltransferase
mTOR: mammalian target of rapamycin
MTT: 3-(4,5-dimethylthiazol-2-yl)-2,5-diphenyltetrazolium bromide
NCBI: National Center for Biotechnology Information
p70S6K: p70 ribosomal protein S6 kinase
PBS: phosphate-buffered saline
PI3K: phosphatidylinositol 3-kinase
Raptor: regulatory associated protein of MTOR
SAPK: stress-activated kinases
SDS: sodium dodecyl sulfate
TCTP: translationally controlled tumor protein
TMZ: temozolomide
VEGF: vascular endothelial growth factor
