# Supplementary material for: Identification of thioridazine, an antipsychotic drug, as an antiglioblastoma and anticancer stem cell agent using public gene expression data
Source: Cell Death Dis. 2015 May 7;6(5):e1753–. doi: 10.1038/cddis.2015.77 (PMC4669717; doi:10.1038/cddis.2015.77)
Supplement: Supplementary Information [file cddis201577x1.pdf]

Supplementary Figures and Tables

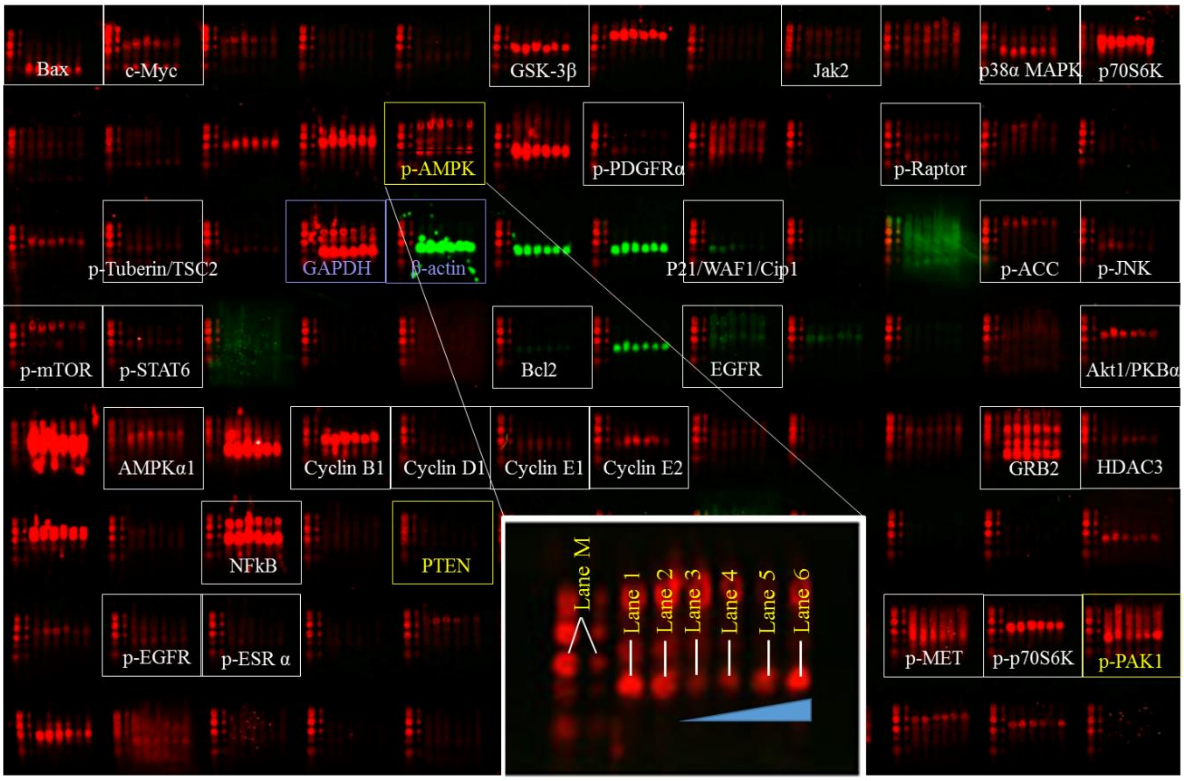

Supplementary information, Figure S1. Identification of differential protein expressions from thioridazine and fluphenazine-treated GBM8401 cells *via* microwestern. GBM8401 cells were treated with various drugs for 24 h. Sample serials (each block from left to right): Lane M (Marker), Lane 1 (fluphenazine 20 μM) and Lane 2 (10 μM), Lane 3 (Control), Lane 4 (thioridazine 5 μM), Lane 5 (10 μM), and Lane 6 (15 μM). GAPDH and β-actin as internal control.

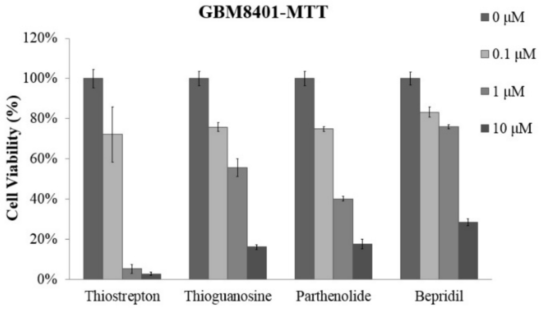

Supplementary information, Figure S2. GBM8401 cells were treated with thiostrepton, thioguanosine, parthenolide, and bepridil at concentrations ranging from 0.1 to 10 μM, and the cell viability was determined using an MTT assay.

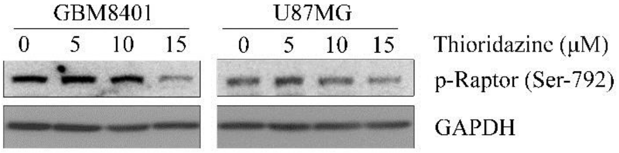

Supplementary information, Figure S3. Thioridazine decreased the expression of phospho-Raptor (Ser-792) in GBM cells. GBM8401 and U87MG cells were treated with thioridazine at 5, 10 and 15 μM for 24 h. Phospho-Raptor (Ser-792) was detected by western blotting. GAPDH was used as an internal control.

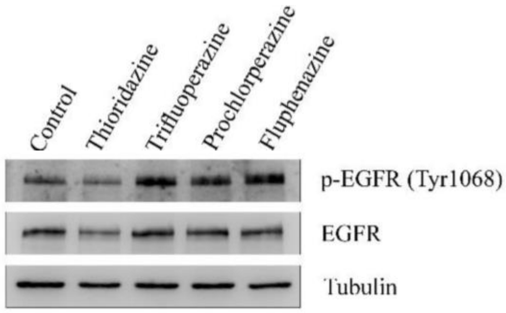

Supplementary information, Figure S4. Fluphenazine increased the expression of phospho-epidermal growth factor receptor (p-EGFR) (Tyr-1068) in GBM cells. GBM8401 cells were treated with fluphenazine at 10 μM for 24 h. Phospho-EGFR (Tyr-1068) was detected by western blotting. Tubulin was used as an internal control.

Supplementary information, Table S1. Two hundred fifteen drugs predicted by Cmap and the result of tested drugs in GBM8401. If the IC_{50} value of the drug was less than 10 μM, we defined the drug as an “effective” drug. (“v”: effective in GBM cells. “x”: ineffective in GBM cells. “ND”: not determined.)

Drug name	Frequency	Result	Anti-psychotic agent
1,4-chrysenequinone	100%	ND	
0173570-0000	100%	ND	
Repaglinide	80%	x	
Procaine	80%	x	
Pargyline	80%	x	
Luteolin	80%	v	
Gliclazide	80%	x	
Chrysin	80%	x	
Tyloxapol	60%	v	
Thiostrepton	60%	v	
Sulfamethoxypyridazine	60%	ND	
Skimmianine	60%	x	
Ronidazole	60%	x	
Phthalylsulfathiazole	60%	x	
Pentoxyverine	60%	ND	
Omeprazole	60%	ND	
Nipecotic acid	60%	ND	
Meticrane	60%	x	
Meropenem	60%	ND	
Medrysone	60%	x	
Levamisole	60%	ND	
Ginkgolide A	60%	x	
Dipyridamole	60%	x	
Apigenin	60%	x	
Prestwick-665	60%	x	
Prestwick-559	60%	x	
DL-thiorphan	60%	x	
Zomepirac	40%	ND	
Vorinostat	40%	v	
Verteporfin	40%	v	
Triflusal	40%	x	
Tridihexethyl	40%	ND	
Trichostatin A	40%	v	
Trazodone	40%	x	
Thioguanosine	40%	v	
Terguride	40%	ND	
Sulfametoxydiazine	40%	x	

Sulconazole	40%	v	
Scopolamine N-oxide	40%	ND	
Riboflavin	40%	ND	
Resveratrol	40%	x	
Piperlongumine	40%	v	
Phenoxybenzamine	40%	v	
Morantel	40%	x	
Monobenzone	40%	ND	
Methazolamide	40%	ND	
Menadione	40%	ND	
Lysergol	40%	ND	
Ketanserin	40%	ND	
Imipenem	40%	ND	
Heliotrine	40%	ND	
Fipexide	40%	ND	
Eucatropine	40%	x	
Ellipticine	40%	v	
Diltiazem	40%	x	
Chlorzoxazone	40%	ND	
Carbachol	40%	ND	
Caffeic acid	40%	ND	
Bisacodyl	40%	ND	
Betaxolol	40%	ND	
Bepridil	40%	v	
Antimycin A	40%	v	
Alsterpaullone	40%	ND	
Alpha-estradiol	40%	x	
Acepromazine	40%	x	o
Prestwick-682	40%	ND	
Prestwick-1084	40%	x	
PNU-0230031	40%	ND	
PHA-00851261E	40%	ND	
N-acetyl-L-leucine	40%	ND	
GW-8510	40%	v	
8-azaguanine	40%	v	
0297417-0002B	40%	ND	
Zimeldine	20%	x	
Yohimbic acid	20%	ND	
Velnacrine	20%	ND	
Valproic acid	20%	ND	
Ursodeoxycholic acid	20%	ND	
Trimetazidine	20%	ND	
Trifluridine	20%	ND	
Trifluoperazine	20%	v	o
Tretinoin	20%	ND	

Tolfenamic acid	20%	ND	
Tolbutamide	20%	ND	
Tobramycin	20%	ND	
Tinidazole	20%	ND	
Tiabendazole	20%	ND	
Thioridazine	20%	v	o
Thioperamide	20%	ND	
Thiamazole	20%	ND	
Thalidomide	20%	ND	
Tanespimycin	20%	v	
Talampicillin	20%	ND	
Sulfamethizole	20%	ND	
Streptozocin	20%	ND	
Streptomycin	20%	ND	
Spiradoline	20%	ND	
Solasodine	20%	ND	
Semustine	20%	x	
Scriptaid	20%	ND	
Roxarsone	20%	ND	
Rottlerin	20%	ND	
Rimexolone	20%	ND	
Quinostatin	20%	ND	
Quinethazone	20%	ND	
Pyrvinium	20%	v	
Puromycin	20%	ND	
Pseudopelletierine	20%	ND	
Proxyphylline	20%	ND	
Propylthiouracil	20%	ND	
Propafenone	20%	v	
Prochlorperazine	20%	v	o
Piroxicam	20%	ND	
Piperidolate	20%	ND	
Piperacetazine	20%	ND	o
Pipenzolate bromide	20%	ND	
Phenacetin	20%	ND	
Parthenolide	20%	v	
Paroxetine	20%	ND	
Oxytetracycline	20%	ND	
Oxprenolol	20%	ND	
Novobiocin	20%	ND	
Norcyclobenzaprine	20%	v	
Nizatidine	20%	ND	
Nitrendipine	20%	ND	
Natamycin	20%	ND	

Metyrapone	20%	ND	
Methotrexate	20%	ND	
Mephenesin	20%	ND	
Mepenzolate bromide	20%	ND	
Lymecycline	20%	ND	
Loracarbef	20%	ND	
Lidoflazine	20%	ND	
Levonorgestrel	20%	x	
Karakoline	20%	ND	
Ifosfamide	20%	ND	
Hexestrol	20%	x	
Hexamethonium bromide	20%	ND	
Harmol	20%	ND	
Harmine	20%	ND	
Guanethidine	20%	ND	
Guaifenesin	20%	ND	
Glibenclamide	20%	ND	
Fusaric acid	20%	ND	
Fluspirilene	20%	x	o
Fluorometholone	20%	ND	
Flunarizine	20%	ND	
Flumetasone	20%	ND	
Flecainide	20%	ND	
Flavoxate	20%	ND	
Famprofazone	20%	ND	
Etomidate	20%	ND	
Etofenamate	20%	ND	
Ethambutol	20%	ND	
Etanidazole	20%	ND	
Estriol	20%	x	
Emetine	20%	v	
Ebselen	20%	ND	
Doxorubicin	20%	ND	
Dirithromycin	20%	ND	
Dioxybenzone	20%	ND	
Dinoprost	20%	ND	
Dimenhydrinate	20%	ND	
Dihydroergocristine	20%	ND	
Dicoumarol	20%	ND	
Dexpropranolol	20%	ND	
Dequalinium chloride	20%	ND	
Deptropine	20%	ND	
Daunorubicin	20%	v	
Dacarbazine	20%	ND	
Cycloserine	20%	v	

Cromoglicic acid	20%	ND	
Cortisone	20%	ND	
Cloperastine	20%	v	
Clomipramine	20%	ND	
Cicloheximide	20%	ND	
Chlorpromazine	20%	v	o
Cefalonium	20%	ND	
Cefalexin	20%	ND	
Carbamazepine	20%	ND	
Canrenoic acid	20%	ND	
Canavanine	20%	ND	
Camptothecin	20%	v	
Bufexamac	20%	ND	
Bromopride	20%	ND	
Bephenium hydroxynaphthoate	20%	ND	
Bemegride	20%	ND	
Atropine	20%	ND	
Atovaquone	20%	ND	
Arachidonic acid	20%	ND	
Apramycin	20%	ND	
Amylocaine	20%	ND	
Amrinone	20%	ND	
Amoxapine	20%	x	
Amiodarone	20%	v	
Alfaxalone	20%	ND	
Acebutolol	20%	ND	
Y-27632	20%	ND	
S-propranolol	20%	ND	
Prestwick-860	20%	ND	
Prestwick-685	20%	v	
Prestwick-674	20%	ND	
Prestwick-664	20%	ND	
MS-275	20%	ND	
LY-294002	20%	ND	
H-7	20%	ND	
CP-690334-01	20%	ND	
CP-320650-01	20%	ND	
6-bromoindirubin-3'-oxime	20%	ND	
5707885	20%	ND	
4,5-dianilinophthalimide	20%	ND	
3-acetylcoumarin	20%	ND	
15-delta prostaglandin J2	20%	v	
0179445-0000	20%	ND	
0175029-0000	20%	ND	

Supplementary information, Table S2. ConsensusPathDB analysis of common GBM differentially expressed probesets with at least 3 appearances from 5 GBM datasets.

pathway name (up-regulated genes)	set size	candidates contained	p-value	q-value	pathway source
Beta1 integrin cell surface interactions	66	7 (10.6%)	1.57E-08	2.17E-06	PID
Beta3 integrin cell surface interactions	44	5 (11.4%)	1.47E-06	8.55E-05	PID
Platelet aggregation inhibitor pathway, pharmacodynamics	49	5 (10.2%)	2.54E-06	8.55E-05	PharmGKB
Intrinsic prothrombin activation pathway	23	4 (17.4%)	3.10E-06	8.55E-05	PID
Intrinsic prothrombin activation pathway	23	4 (17.4%)	3.10E-06	8.55E-05	BioCarta
Complement and coagulation cascades	54	5 (9.3%)	4.14E-06	9.51E-05	Wikipathways
Integrins in angiogenesis	65	5 (7.8%)	9.64E-06	0.00019	PID
Complement and coagulation cascades-*Homo sapiens* (human)	69	5 (7.2%)	1.40E-05	0.000241	KEGG
NCAM signaling for neurite out-growth	71	5 (7.0%)	1.61E-05	0.000247	Reactome
Muscle cell TarBase	354	9 (2.6%)	1.86E-05	0.000257	Wikipathways
Extrinsic prothrombin activation pathway	13	3 (23.1%)	2.43E-05	0.00028	PID
Extrinsic prothrombin activation pathway	13	3 (23.1%)	2.43E-05	0.00028	BioCarta
Focal adhesion-*Homo sapiens* (human)	200	7 (3.5%)	2.93E-05	0.000311	KEGG
Epithelium TarBase	290	8 (2.8%)	3.56E-05	0.000351	Wikipathways
ECM-receptor interaction-*Homo sapiens* (human)	85	5 (5.9%)	3.86E-05	0.000356	KEGG
PLK1 signaling events	44	4 (9.1%)	4.43E-05	0.000359	PID
NCAM1 interactions	44	4 (9.1%)	4.43E-05	0.000359	Reactome
Amoebiasis-*Homo sapiens* (human)	108	5 (4.7%)	0.000111	0.000853	KEGG
Focal adhesion	184	6 (3.3%)	0.000169	0.00123	Wikipathways
Signaling by PDGF	125	5 (4.0%)	0.000233	0.00153	Reactome
Integrin	125	5 (4.0%)	0.000233	0.00153	INOH
Inflammatory response pathway	32	3 (9.4%)	0.000397	0.00238	Wikipathways
Syndecan-4-mediated signaling	32	3 (9.4%)	0.000397	0.00238	PID

events					
Protein digestion and absorption-*Homo sapiens* (human)	81	4 (4.9%)	0.00048	0.00276	KEGG
Integrin cell surface interactions	85	4 (4.7%)	0.000577	0.00318	Reactome
Diabetes pathways	39	3 (7.9%)	0.000662	0.00352	Reactome
APC/C-mediated degradation of cell cycle proteins	10	2 (20.0%)	0.000888	0.00438	Wikipathways
Regulation of insulin-like growth factor (IGF) activity by insulin-like growth factor binding proteins (IGFBPs)	10	2 (20.0%)	0.000888	0.00438	Wikipathways
Regulators of bone mineralization	11	2 (18.2%)	0.00108	0.00485	PID
Regulators of bone mineralization	11	2 (18.2%)	0.00108	0.00485	BioCarta
Syndecan-1-mediated signaling events	45	3 (6.7%)	0.00109	0.00485	PID
Axon guidance	268	6 (2.3%)	0.0012	0.00518	Reactome
Regulation of insulin-like growth factor (IGF) activity by insulin-like growth factor binding proteins (IGFBPs)	12	2 (16.7%)	0.0013	0.00542	PID
GRB2 events in EGFR signaling	14	2 (14.3%)	0.00178	0.00681	Reactome
Regulation of complement cascade	14	2 (14.3%)	0.00178	0.00681	Reactome
Nicotinate and nicotinamide metabolism	14	2 (14.3%)	0.00178	0.00681	SMPDB
SHC1 events in EGFR signaling	15	2 (13.3%)	0.00204	0.00762	Reactome
Celecoxib pathway, pharmacodynamics	58	3 (5.2%)	0.00227	0.00826	PharmGKB
Platelet activation, signaling and aggregation	215	5 (2.4%)	0.00261	0.00908	Reactome
Regulation of insulin-like growth factor (IGF) activity by insulin-like growth factor binding proteins (IGFBPs)	17	2 (11.8%)	0.00263	0.00908	Reactome
Lymphocyte TarBase	437	7 (1.6%)	0.00286	0.00962	Wikipathways
Nucleotide metabolism	19	2 (10.5%)	0.00329	0.0106	Wikipathways
Pathways in cancer-*Homo sapiens* (human)	325	6 (1.8%)	0.00331	0.0106	KEGG
p53 signaling pathway-*Homo sapiens* (human)	68	3 (4.4%)	0.00358	0.0112	KEGG
Formation of fibrin clot	20	2 (10.0%)	0.00364	0.0112	Wikipathwa

(clotting cascade)					ys
GRB2 events in ERBB2 signaling	23	2 (8.7%)	0.00481	0.0141	Reactome
Nicotinate nicotinamide metabolism	23	2 (8.7%)	0.00481	0.0141	INOH
Complement cascade	78	3 (3.9%)	0.00489	0.0141	Reactome
Developmental Biology	363	6 (1.7%)	0.00545	0.0151	Reactome
SHC1 events in ERBB2 signaling	25	2 (8.0%)	0.00567	0.0151	Reactome
VEGFR3 signaling in lymphatic endothelium	25	2 (8.0%)	0.00567	0.0151	PID
Alpha9 beta1 integrin signaling events	25	2 (8.0%)	0.00567	0.0151	PID
Nicotinate and nicotinamide metabolism-*Homo sapiens* (human)	26	2 (7.7%)	0.00613	0.016	KEGG
Platelet degranulation	85	3 (3.6%)	0.00626	0.016	Reactome
Small cell lung cancer-*Homo sapiens* (human)	84	3 (3.6%)	0.00647	0.016	KEGG
Cdc20: phospho-APC/C mediated degradation of cyclin A	29	2 (7.4%)	0.0066	0.016	Reactome
APC/C: Cdc20 mediated degradation of mitotic proteins	29	2 (7.4%)	0.0066	0.016	Reactome
Activation of APC/C and APC/C: Cdc20 mediated degradation of mitotic proteins	30	2 (7.1%)	0.00708	0.0167	Reactome
Mitotic prometaphase	87	3 (3.4%)	0.00713	0.0167	PID
Response to elevated platelet cytosolic Ca^{2+}	90	3 (3.4%)	0.00736	0.0169	Reactome
Gap junction-*Homo sapiens* (human)	89	3 (3.4%)	0.00759	0.0172	KEGG
Mitotic prometaphase	95	3 (3.2%)	0.00908	0.0201	Reactome
Formation of fibrin clot (clotting cascade)	32	2 (6.2%)	0.00919	0.0201	Reactome
Cell surface interactions at the vascular wall	99	3 (3.1%)	0.00934	0.0201	Reactome

pathway name (down-regulated genes)	set size	candidates contained	p-value	q-value	pathway source
Transmission across chemical synapses	195	20 (10.3%)	4.66E-14	1.93E-11	Reactome
Neuronal system	288	23 (8.0%)	1.07E-13	2.22E-11	Reactome
GABAergic synapse-*Homo sapiens* (human)	89	14 (15.7%)	1.05E-12	1.45E-10	KEGG
Glutamatergic synapse-*Homo sapiens* (human)	126	13 (10.3%)	1.61E-09	1.67E-07	KEGG
Neurotransmitter release cycle	36	8 (22.2%)	5.20E-09	4.32E-07	Reactome

Morphine addiction-*Homo sapiens* (human)	92	11 (12.0%)	6.49E-09	4.49E-07	KEGG
Synaptic vesicle cycle-*Homo sapiens* (human)	64	9 (14.1%)	3.89E-08	2.30E-06	KEGG
Neurotransmitter receptor binding and downstream transmission in the postsynaptic cell	141	12 (8.5%)	6.29E-08	3.26E-06	Reactome
Calcium regulation in the cardiac cell	149	12 (8.1%)	1.16E-07	5.35E-06	Wikipathways
Retrograde endocannabinoid signaling-*Homo sapiens* (human)	102	10 (9.8%)	2.22E-07	9.22E-06	KEGG
Glutamate neurotransmitter release cycle	16	5 (31.2%)	7.00E-07	2.64E-05	Reactome
GABA synthesis, release, reuptake and degradation	19	5 (26.3%)	1.81E-06	6.27E-05	Reactome
GABA receptor activation	55	7 (12.7%)	2.70E-06	8.63E-05	Reactome
Glutamate neurotransmitter release cycle	11	4 (36.4%)	4.99E-06	0.000148	PID
Serotonin neurotransmitter release cycle	12	4 (33.3%)	7.42E-06	0.000193	Reactome
Dopamine neurotransmitter release cycle	12	4 (33.3%)	7.42E-06	0.000193	Reactome
G protein signaling pathways	92	8 (8.7%)	9.42E-06	0.00021	Wikipathways
Signaling pathway from G protein families	26	5 (19.2%)	9.63E-06	0.00021	PID
signaling pathway from G protein families	26	5 (19.2%)	9.63E-06	0.00021	BioCarta
L1CAM interactions	97	8 (8.2%)	1.40E-05	0.000268	Reactome
Recycling pathway of L1	28	5 (17.9%)	1.41E-05	0.000268	Reactome
G alpha (z) signalling events	47	6 (12.8%)	1.42E-05	0.000268	Reactome
Endocrine and other factor-regulated calcium reabsorption-*Homo sapiens* (human)	49	6 (12.2%)	1.81E-05	0.000327	KEGG
Neurotransmitter release cycle	15	4 (26.7%)	1.99E-05	0.000333	Wikipathways
Gastric acid secretion-*Homo sapiens* (human)	74	7 (9.5%)	2.01E-05	0.000333	KEGG
Endocytotic role of ndk phosphins and dynamin	17	4 (23.5%)	3.42E-05	0.000525	PID
Endocytotic role of ndk phosphins and dynamin	17	4 (23.5%)	3.42E-05	0.000525	BioCarta
Cholinergic synapse-*Homo sapiens* (human)	112	8 (7.1%)	3.99E-05	0.000591	KEGG
Axon guidance	268	12 (4.5%)	4.89E-05	0.0007	Reactome
GPCR signaling-G alpha s	272	12 (4.4%)	6.07E-05	0.00084	INOH

Epac and ERK					
Gap junction-*Homo sapiens* (human)	89	7 (7.9%)	6.67E-05	0.000866	KEGG
Myometrial relaxation and contraction pathways	155	9 (5.8%)	6.68E-05	0.000866	Wikipathways
Hemostasis	471	16 (3.4%)	7.31E-05	0.000919	Reactome
Endothelins	65	6 (9.4%)	8.47E-05	0.000978	PID
Activation of GABAB receptors	40	5 (12.5%)	8.48E-05	0.000978	Reactome
GABA B receptor activation	40	5 (12.5%)	8.48E-05	0.000978	Reactome
GPCR signaling-G alpha s PKA and ERK	285	12 (4.2%)	9.50E-05	0.00107	INOH
Endocytosis-*Homo sapiens* (human)	202	10 (5.0%)	0.0001	0.00107	KEGG
Beta-agonist/beta-blocker pathway, pharmacodynamics	66	6 (9.1%)	0.000101	0.00107	PharmGKB
Dopaminergic synapse-*Homo sapiens* (human)	130	8 (6.2%)	0.000115	0.00109	KEGG
Effects of Botulinum toxin	9	3 (33.3%)	0.000116	0.00109	PID
Dopamine neurotransmitter release cycle	9	3 (33.3%)	0.000116	0.00109	PID
Neurotransmitter release cycle	9	3 (33.3%)	0.000116	0.00109	PID
Serotonin neurotransmitter release cycle	9	3 (33.3%)	0.000116	0.00109	PID
GABA synthesis	2	2 (100.0%)	0.000129	0.00117	Reactome
Glutamate dependent acid resistance	2	2 (100.0%)	0.000129	0.00117	HumanCyc
Long-term depression-*Homo sapiens* (human)	70	6 (8.6%)	0.00014	0.00121	KEGG
Regulation of ck1/cdk5 by type 1 glutamate receptors	24	4 (16.7%)	0.000143	0.00121	PID
Regulation of ck1/cdk5 by type 1 glutamate receptors	24	4 (16.7%)	0.000143	0.00121	BioCarta
Ion channels and their functional role in vascular endothelium	47	5 (10.6%)	0.000186	0.00151	BioCarta
Ion channels and their functional role in vascular endothelium	47	5 (10.6%)	0.000186	0.00151	PID
GPCR signaling-G alpha i	262	11 (4.2%)	0.000193	0.00151	INOH
GPCR signaling-pertussis toxin	262	11 (4.2%)	0.000193	0.00151	INOH
Interaction between L1 and Ankyrins	26	4 (15.4%)	0.000198	0.00152	Reactome
Calcium signaling pathway-*Homo sapiens* (human)	183	9 (5.0%)	0.000218	0.00157	KEGG
Acetylcholine neurotransmitter release cycle	11	3 (27.3%)	0.000224	0.00157	Reactome

Acetylcholine neurotransmitter release cycle	11	3 (27.3%)	0.000224	0.00157	PID
Norepinephrine neurotransmitter release cycle	11	3 (27.3%)	0.000224	0.00157	Reactome
Norepinephrine neurotransmitter release cycle	11	3 (27.3%)	0.000224	0.00157	PID
GPCR signaling-cholera toxin	270	11 (4.1%)	0.000251	0.00173	INOH
Selective serotonin reuptake inhibitor pathway, pharmacodynamics	28	4 (14.3%)	0.000267	0.00181	PharmGKB
GPCR signaling-G alpha q	274	11 (4.0%)	0.000285	0.00191	INOH
Alanine and aspartate metabolism	12	3 (25.0%)	0.000296	0.00195	Wikipathways
Trafficking of AMPA receptors	30	4 (13.3%)	0.000351	0.00224	Reactome
Glutamate binding, activation of AMPA receptors and synaptic plasticity	30	4 (13.3%)	0.000351	0.00224	Reactome
Effects of calcineurin in keratinocyte differentiation	13	3 (23.1%)	0.000381	0.00233	PID
Effects of calcineurin in keratinocyte differentiation	13	3 (23.1%)	0.000381	0.00233	BioCarta
Recycling pathway of L1	13	3 (23.1%)	0.000381	0.00233	PID
Opioid signalling	85	6 (7.1%)	0.000407	0.00245	Reactome
Alanine, aspartate and glutamate metabolism-*Homo sapiens* (human)	32	4 (12.5%)	0.000452	0.00268	KEGG
Serotonergic synapse-*Homo sapiens* (human)	122	7 (5.7%)	0.000477	0.00273	KEGG
S1P4 pathway	15	3 (21.4%)	0.000481	0.00273	PID
Retrograde neurotrophin signalling	14	3 (21.4%)	0.000481	0.00273	Reactome
G alpha (i) signalling events	206	9 (4.4%)	0.000566	0.00317	Reactome
Gamma-aminobutyric acid receptor life cycle pathway	15	3 (20.0%)	0.000596	0.00317	PID
Gamma-aminobutyric acid receptor life cycle pathway	15	3 (20.0%)	0.000596	0.00317	BioCarta
GPCRs, class C metabotropic glutamate, pheromone	15	3 (20.0%)	0.000596	0.00317	Wikipathways
Class C/3 (Metabotropic glutamate/pheromone receptors)	15	3 (20.0%)	0.000596	0.00317	Reactome
Ion channel transport	61	5 (8.2%)	0.000634	0.00333	Reactome
Glutamate degradation III (*via* 4-aminobutyrate)	4	2 (50.0%)	0.000763	0.00396	HumanCyc
Developmental biology	363	12 (3.3%)	0.000805	0.00413	Reactome
Internalization of ErbB1	39	4 (10.5%)	0.000881	0.00446	PID
Integration of energy	101	6 (6.0%)	0.000966	0.00483	Reactome

metabolism					
Melanogenesis-*Homo sapiens* (human)	101	6 (5.9%)	0.00102	0.00497	KEGG
Potassium channels	101	6 (5.9%)	0.00102	0.00497	Reactome
Nicotine addiction-*Homo sapiens* (human)	40	4 (10.0%)	0.00107	0.00517	KEGG
EGF-EGFR signaling pathway	141	7 (5.0%)	0.00113	0.00537	Wikipathways
Long-term potentiation-*Homo sapiens* (human)	70	5 (7.1%)	0.00119	0.00561	KEGG
Nitric oxide signaling pathway	19	3 (15.8%)	0.00123	0.00566	PID
Nitric oxide signaling pathway	19	3 (15.8%)	0.00123	0.00566	BioCarta
Creatine-phosphate biosynthesis	5	2 (40.0%)	0.00126	0.00569	HumanCyc
Transmembrane transport of small molecules	433	13 (3.0%)	0.00128	0.00569	Reactome
Actions of nitric oxide in the heart	42	4 (9.5%)	0.00129	0.00569	BioCarta
Actions of nitric oxide in the heart	42	4 (9.5%)	0.00129	0.00569	PID
PAR1-mediated thrombin signaling events	44	4 (9.1%)	0.00154	0.00671	PID
PLC beta mediated events	45	4 (8.9%)	0.00167	0.00723	Reactome
G protein mediated events	46	4 (8.7%)	0.00182	0.00769	Reactome
Proton pump inhibitor pathway, pharmacodynamics	46	4 (8.7%)	0.00182	0.00769	PharmGKB
Regulation of insulin secretion	80	5 (6.3%)	0.00204	0.00856	Reactome
How progesterone initiates the oocyte maturation	23	3 (13.0%)	0.00217	0.00892	PID
How progesterone initiates the oocyte maturation	23	3 (13.0%)	0.00217	0.00892	BioCarta
Cocaine addiction-*Homo sapiens* (human)	50	4 (8.0%)	0.00247	0.0101	KEGG
Platelet homeostasis	85	5 (6.0%)	0.00267	0.0108	Reactome
Alanine aspartate asparagine metabolism	25	3 (12.0%)	0.00277	0.0111	INOH
Amyotrophic lateral sclerosis (ALS)-*Homo sapiens* (human)	53	4 (7.5%)	0.00306	0.0116	KEGG
TSH	26	3 (11.5%)	0.00311	0.0116	NetPath
Inhibition of insulin secretion by adrenaline/noradrenaline	26	3 (11.5%)	0.00311	0.0116	Reactome
CXCR4-mediated signaling events	89	5 (5.7%)	0.00312	0.0116	PID
Salivary secretion-*Homo sapiens* (human)	89	5 (5.6%)	0.00344	0.0116	KEGG
S1P5 pathway	8	2 (25.0%)	0.00346	0.0116	PID
Axonal growth inhibition (RHOA activation)	8	2 (25.0%)	0.00346	0.0116	Reactome

Organic anion transporters	8	2 (25.0%)	0.00346	0.0116	Reactome
Creatine metabolism	8	2 (25.0%)	0.00346	0.0116	Reactome
Glutamate glutamine metabolism	27	3 (11.1%)	0.00347	0.0116	INOH
Transcription factor creb and its extracellular signals	27	3 (11.1%)	0.00347	0.0116	PID
Transcription factor creb and its extracellular signals	27	3 (11.1%)	0.00347	0.0116	BioCarta
Activation of G protein gated potassium channels	27	3 (11.1%)	0.00347	0.0116	Reactome
G protein gated potassium channels	27	3 (11.1%)	0.00347	0.0116	Reactome
Inhibition of voltage gated Ca^{2+} channels *via* G beta/gamma subunits	27	3 (11.1%)	0.00347	0.0116	Reactome
Basigin interactions	29	3 (11.1%)	0.00347	0.0116	Reactome
L1CAM interactions	27	3 (11.1%)	0.00347	0.0116	Wikipathways
T cell receptor signaling pathway	55	4 (7.3%)	0.00351	0.0116	PID
T cell receptor signaling pathway	55	4 (7.3%)	0.00351	0.0116	BioCarta
Antiarrhythmic pathway, pharmacodynamics	55	4 (7.3%)	0.00351	0.0116	PharmGKB
Arginine proline metabolism	56	4 (7.3%)	0.00351	0.0116	INOH
Tight junction-*Homo sapiens* (human)	132	6 (4.6%)	0.00381	0.0123	KEGG
Thrombin signaling and protease-activated receptors	28	3 (10.7%)	0.00385	0.0123	PID
Thrombin signaling and protease-activated receptors	28	3 (10.7%)	0.00385	0.0123	BioCarta
GPCR Group I metabotropic glutamate receptor	28	3 (10.7%)	0.00385	0.0123	INOH
Nitric oxide stimulates guanylate cyclase	28	3 (10.7%)	0.00385	0.0123	Reactome
G alpha (s) signalling events	132	6 (4.5%)	0.00396	0.0125	Reactome
p75NTR regulates axonogenesis	9	2 (22.2%)	0.00441	0.0139	Reactome
Fc gamma R-mediated phagocytosis-*Homo sapiens* (human)	97	5 (5.3%)	0.00455	0.0141	KEGG
Fmlp induced chemokine gene expression in hmc-1 cells	30	3 (10.0%)	0.0047	0.0141	PID
Fmlp induced chemokine gene expression in hmc-1 cells	30	3 (10.0%)	0.0047	0.0141	BioCarta
Role of mef2d in T-cell apoptosis	30	3 (10.0%)	0.0047	0.0141	PID
Role of mef2d in T-cell apoptosis	30	3 (10.0%)	0.0047	0.0141	BioCarta

G protein activation	30	3 (10.0%)	0.0047	0.0141	Reactome
Nongenotropic androgen signaling	32	3 (9.7%)	0.00516	0.0154	PID
Taurine and hypotaurine metabolism-*Homo sapiens* (human)	10	2 (20.0%)	0.00547	0.0158	KEGG
Regulation of spermatogenesis by crem	10	2 (20.0%)	0.00547	0.0158	PID
Regulation of spermatogenesis by crem	10	2 (20.0%)	0.00547	0.0158	BioCarta
Retrograde neurotrophin signalling	10	2 (20.0%)	0.00547	0.0158	PID
Lysosome vesicle biogenesis	10	2 (20.0%)	0.00547	0.0158	PID
Hypothetical network for drug addiction	32	3 (9.4%)	0.00564	0.0158	Wikipathways
Bcr signaling pathway	32	3 (9.4%)	0.00564	0.0158	BioCarta
Bcr signaling pathway	32	3 (9.4%)	0.00564	0.0158	PID
Signaling by robo receptor	33	3 (9.4%)	0.00564	0.0158	Reactome
GnRH signaling pathway-*Homo sapiens* (human)	101	5 (5.0%)	0.00589	0.0163	KEGG
Pancreatic secretion-*Homo sapiens* (human)	101	5 (5.0%)	0.00589	0.0163	KEGG
Phospholipids as signalling intermediaries	33	3 (9.1%)	0.00616	0.0164	PID
Phospholipids as signalling intermediaries	33	3 (9.1%)	0.00616	0.0164	BioCarta
Bioactive peptide induced signaling pathway	33	3 (9.1%)	0.00616	0.0164	PID
Bioactive peptide induced signaling pathway	33	3 (9.1%)	0.00616	0.0164	BioCarta
Inwardly rectifying K^+ channels	33	3 (9.1%)	0.00616	0.0164	Reactome
Regulation of Ras family activation	33	3 (9.1%)	0.00616	0.0164	PID
Glucagon signaling in metabolic regulation	35	3 (8.6%)	0.00727	0.019	Reactome
Post NMDA receptor activation events	35	3 (8.6%)	0.00727	0.019	Reactome
Endothelin	35	3 (8.6%)	0.00727	0.019	Wikipathways
Purine nucleotides nucleosides metabolism	109	5 (4.6%)	0.00778	0.0202	INOH
Ion transport by P-type ATPases	36	3 (8.3%)	0.00786	0.0203	Reactome
GABA A receptor activation	12	2 (16.7%)	0.00791	0.0203	Reactome
Amphetamine addiction-*Homo sapiens* (human)	70	4 (5.7%)	0.00828	0.0211	KEGG
G13 signaling pathway	37	3 (8.1%)	0.00849	0.0215	Wikipathways

CXCR3-mediated signaling events	38	3 (7.9%)	0.00914	0.0221	PID
Intracellular signalling through adenosine receptor A2a and adenosine	38	3 (7.9%)	0.00914	0.0221	SMPDB
Intracellular signalling through adenosine receptor A2b and adenosine	38	3 (7.9%)	0.00914	0.0221	SMPDB
Regulation of insulin secretion by glucagon-like peptide-1	38	3 (7.9%)	0.00914	0.0221	Reactome
Aspartate metabolism	13	2 (15.4%)	0.00928	0.0221	SMPDB
Adenylate cyclase inhibitory pathway	13	2 (15.4%)	0.00928	0.0221	Reactome
Inhibition of adenylate cyclase pathway	13	2 (15.4%)	0.00928	0.0221	Reactome
Attenuation of GPCR signaling	13	2 (15.4%)	0.00928	0.0221	PID
Attenuation of GPCR signaling	13	2 (15.4%)	0.00928	0.0221	BioCarta
D-myo-inositol (1,4,5)-trisphosphate degradation	13	2 (15.4%)	0.00928	0.0221	HumanCyc
Activation of NMDA receptor upon glutamate binding and postsynaptic events	39	3 (7.7%)	0.00982	0.0233	Reactome

Supplementary information, Table S3. Antibodies used in the microwestern assay.

**A1**	Bax antibody
**A2**	c-Myc (D84C12) XP® rabbit mAb
**A3**	c-Raf antibody
**A4**	FAK antibody
**A5**	FGF receptor 1 antibody
**A6**	GSK-3β (27C10) rabbit mAb
**A7**	HSP90 antibody
**A8**	Jak1 (6G4) rabbit mAb
**A9**	Jak2 (D2E12) XP® rabbit mAb
**A10**	mTOR (7C10) rabbit mAb
**A11**	p38α MAPK (7D6) rabbit mAb
**A12**	p70 S6 kinase (49D7) rabbit mAb
**B1**	PLCγ2 antibody
**B2**	Rb (D20) rabbit mAb
**B3**	SAPK/JNK (56G8) rabbit mAb
**B4**	Src (36D10) rabbit mAb
**B5**	Phospho-AMPKα (Thr172) (D79.5E) rabbit mAb
**B6**	Phospho-p38 MAPK (Thr180/Tyr182) (D3F9) XP™ rabbit mAb
**B7**	Phospho-PDGF receptor α (Tyr1018) antibody
**B8**	Phospho-PDGF receptor β (Tyr1009) (42F9) rabbit mAb
**B9**	Phospho-PLCγ1 (Tyr783) antibody
**B10**	Phospho-Raptor (Ser792) antibody
**B11**	Phospho-Rb (Ser780) (C84F6) rabbit mAb
**B12**	Phospho-SAPK/JNK (Thr183/Tyr185) (81E11) rabbit mAb
**C1**	Phospho-Src (Tyr527) antibody
**C2**	Phospho-Tuberin/TSC2 (Thr1462) (5B12) rabbit mAb
**C3**	Phospho-VEGF receptor 2 (Tyr1059) (D5A6) rabbit mAb

**E1**	Alpha-tubulin
**E2**	AMPKα1, rabbit monoclonal
**E3**	Cdk4
**E4**	Cyclin B1 (C-term), clone Y106, rabbit monoclonal
**E5**	Cyclin D1, clone EP272Y, rabbit monoclonal
**E6**	Cyclin E1, clone EP435E rabbit monoclonal
**E7**	Cyclin E2, clone EP454Y, rabbit monoclonal
**E8**	ErbB-2/HER-2
**E9**	Estrogen receptor α, clone E115
**E10**	Frizzled 5
**E11**	GRB2
**E12**	HDAC3, clone Y415, rabbit monoclonal; 100 μL
**F1**	HIF-1α
**F2**	MDM2
**F3**	NF- B p50, clone E381
**F4**	PI3 kinase, p110α rabbit monoclonal
**F5**	PI3 kinase, p85, N-SH2, clone UB93-3
**F6**	PTEN rabbit monoclonal
**F7**	Phospho-erbB2 (Thr686), clone 7F8
**F8**	Phospho-GSK3 (Tyr279/Tyr216), clone 5G-2F
**F9**	Phospho-GSK3β (Ser9), clone 2D3
**F10**	Phospho-insulin receptor (Tyr 1150/1151), clone 10C3
**F11**	Phospho-Cdc25C (Ser216), clone E190, rabbit monoclonal
**F12**	Phospho-c-Jun (Ser63),clone Y172; 100 μL
**G1**	Phospho-c-Myc (Thr58/Ser62)
**G2**	Phospho-EGFR (Tyr1086)
**G3**	Phospho-estrogen receptor α (Ser167)

**C4**	GAPDH		**G4**	Phospho-FAK (Tyr861)
**C5**	Actin, near a.a. 50-70, clone C4		**G5**	Phospho-IRS1 (Ser307); 200 µL
**C6**	APC, C-terminus, clone C-APC 28.9		**G6**	Phospho-JAK1 (Tyr1022/Tyr1023)
**C7**	CrkL, clone 5-6; 100 µg		**G7**	Phospho-JAK2 (Tyr1007/1008)
**C8**	p21/WAF1/Cip1, clone CP74		**G8**	Phospho-MAP kinase1/2 (Erk1/2)
**C9**	β-Catenin, clone 2H4A7		**G9**	Phospho-MEK1 (Ser218/222)/MEK2 (Ser222/226)
**C10**	Phospho-ATM (Ser1981), clone 10H11.E12		**G10**	Phospho-Met (Tyr1003)
**C11**	Phospho-acetyl CoA carboxylase (Ser79); 200 µg		**G11**	Phospho-p70 S6 kinase (Thr389), rabbit monoclonal
**C12**	Phospho-JNK (Thr183/Tyr185, Thr221/Tyr223)		**G12**	Phospho-PAK1 (Ser199/Ser204)
**D1**	Phospho-mTOR (Ser2481); 100 µL		**H1**	Phospho-PKA, RII (Ser96), rabbit monoclonal
**D2**	Phospho-STAT6 (Tyr641)		**H2**	Phospho-PKCε (Ser729)
**D3**	Phospho-Akt1/PKBα (Ser473), clone 11E6		**H3**	Phospho-PKD (Ser916)
**D4**	Phospho-MKK4 (Ser257/Thr261)		**H4**	Phospho-PPARγ (Ser82), clone AW504
**D5**	Phospho-PKCδ (Ser645)		**H5**	Phospho-PTEN (Ser385)
**D6**	Bcl2, clone 100		**H6**	Phospho-Src (Tyr416), clone 9A6
**D7**	Cyclin A, clone BF 683		**H7**	Phospho-c-Raf (Ser338/Tyr340)
**D8**	EGFR, clone LA22		**H8**	Phospho-Raf-1 (Tyr340/Tyr341)
**D9**	p53, clone BP53-12		**H9**	Phospho-Src (Tyr418)
**D10**	PI3 Kinase, p85, N-SH3, clone AB6		**H10**	Phospho-STAT1 (Ser727)
**D11**	Acetyl CoA carboxylase 1, rabbit mAb		**H11**	Phospho-STAT2 (Tyr689)
**D12**	Akt1/PKBα, clone AW24		**H12**	Phospho-STAT5A/B (Tyr694/699), clone A11W

Supplementary information, Table S4. GBM differentially expressed probesets with at least 4 appearances from 5 GBM datasets.

probeset (up-regulated)	gene	times
211980_at	COL4A1	5
201761_at	MTHFD2	5
219918_s_at	ASPM	4
219410_at	TMEM45A	4
219148_at	PBK	4
218883_s_at	MLF1IP	4
218678_at	NES	4
218585_s_at	DTL	4
218039_at	NUSAP1	4
215076_s_at	COL3A1	4
211964_at	COL4A2	4
210809_s_at	POSTN	4
206157_at	PTX3	4
205347_s_at	TMSB15A	4
204915_s_at	SOX11	4
203820_s_at	IGF2BP3	4
203819_s_at	IGF2BP3	4
203548_s_at	LPL	4
203358_s_at	EZH2	4
203213_at	CDK1	4
202503_s_at	KIAA0101	4
202404_s_at	COL1A2	4
202403_s_at	COL1A2	4
202007_at	NID1	4
201984_s_at	EGFR	4
201983_s_at	EGFR	4
201664_at	SMC4	4
201617_x_at	CALD1	4
201292_at	TOP2A	4

probeset (down-regulated)	gene	times
221802_s_at	KIAA1598	5
213385_at	CHN2	5
212681_at	EPB41L3	5
209576_at	GNAI1	5
205814_at	GRM3	5
205637_s_at	SH3GL3	5
204681_s_at	RAPGEF5	5
204547_at	RAB40B	5
222005_s_at	GNG3	4
221916_at	NEFL	4
221805_at	NEFL	4
219685_at	TMEM35	4
219671_at	HPCAL4	4
219659_at	ATP8A2	4
219619_at	DIRAS2	4
219368_at	NAP1L2	4
218623_at	HMP19	4
214762_at	ATP6V1G2	4
214434_at	HSPA12A	4
214157_at	GNAS	4
214111_at	OPCML	4
214046_at	FUT9	4
213938_at	ERC2	4
213904_at	---	4
213558_at	PCLO	4
213486_at	COPG2IT1	4
213268_at	CAMTA1	4
213131_at	OLFM1	4
213122_at	TSPYL5	4
213106_at	ATP8A1	4
212624_s_at	CHN1	4
212473_s_at	MICAL2	4
212339_at	EPB41L1	4
211776_s_at	EPB41L3	4
211458_s_at	GABARAPL1 /// GABARAPL3	4
211276_at	TCEAL2	4

210657_s_at	SEPT4	4
210404_x_at	CAMK2B	4
210222_s_at	RTN1	4
210016_at	MYT1L	4
209990_s_at	GABBR2	4
209956_s_at	CAMK2B	4
209839_at	DNM3	4
209755_at	NMNAT2	4
209685_s_at	PRKCB	4
209392_at	ENPP2	4
209242_at	PEG3	4
209072_at	MBP	4
207957_s_at	PRKCB	4
207093_s_at	OMG	4
207010_at	GABRB1	4
206710_s_at	EPB41L3	4
206385_s_at	ANK3	4
206349_at	LGI1	4
206196_s_at	RUNDC3A	4
205818_at	DBC1	4
205795_at	NRXN3	4
205751_at	SH3GL2	4
205737_at	KCNQ2	4
205591_at	OLFM1	4
205352_at	SERPINI1	4
205348_s_at	DYNC1I1	4
205278_at	GAD1	4
205110_s_at	FGF13	4
204995_at	CDK5R1	4
204953_at	SNAP91	4
204823_at	NAV3	4
204793_at	GPRASP1	4
204777_s_at	MAL	4
204743_at	TAGLN3	4
204720_s_at	DNAJC6	4
204684_at	NPTX1	4
204584_at	L1CAM	4
204466_s_at	SNCA	4
204465_s_at	INA	4
203999_at	SYT1	4
203798_s_at	VSNL1	4
203797_at	VSNL1	4
203607_at	INPP5F	4
203485_at	RTN1	4

203001_s_at	STMN2	4
203000_at	STMN2	4
202890_at	MAP7	4
202508_s_at	SNAP25	4
202507_s_at	SNAP25	4
202391_at	BASP1	4
202363_at	SPOCK1	4
202260_s_at	STXBP1	4

Supplementary information, Table S5. Appearance of phenothiazines from the Cmap analysis of three anti-cancer stem cell signatures.

	Drug Name	Rank	Mean	N	Enrichment	*P*
Wong et al. study	Trifluoperazine	1	-0.533	16	-0.579	0
	Prochlorperazine	2	-0.538	16	-0.577	0
	Thioridazine	3	-0.558	20	-0.534	0
	Chlorpromazine	17	-0.402	19	-0.393	0.00389
	Fluphenazine	21	-0.494	18	-0.392	0.00576
	Acepromazine	60	-0.59	4	-0.659	0.0306
	Promethazine	75	-0.582	4	-0.625	0.04884
	Perphenazine	--	--	--	--	--
Shats et al. study	Trifluoperazine	5	-0.342	16	-0.497	0.00028
	Prochlorperazine	9	-0.329	16	-0.475	0.00082
	Thioridazine	13	-0.335	20	-0.401	0.00189
	Chlorpromazine	--	--	--	--	--
	Fluphenazine	43	-0.291	18	-0.266	0.04351
	Acepromazine	--	--	--	--	--
	Promethazine	--	--	--	--	--
	Perphenazine	37	-0.453	5	-0.589	0.03446
Suvà et al. study	Trifluoperazine	47	-0.371	16	-0.417	0.00499
	Prochlorperazine	--	--	--	--	--
	Thioridazine	11	-0.407	20	-0.51	0
	Chlorpromazine	--	--	--	--	--
	Fluphenazine	--	--	--	--	--
	Acepromazine	--	--	--	--	--
	Promethazine	--	--	--	--	--
	Perphenazine	--	--	--	--	--

*(top) The dataset of different gene signatures of ESC and adult stem cells from Wong et al. (2008) [25] is available online (http://www.ncbi.nlm.nih.gov/geo/; [GEO Series accession number GSE10423]). Phenothiazine-like anti-psychotics appear frequently when the Cmap drug list is queried with differential ESC-like to adult stem-like cell gene signatures. There are a total of 77 potential drugs with negative enrichments and $p < 0.05$. Ranking refers to these 77 potential drugs.

(middle) Shats et al. (2011) [23] study used multiple cancer cell lines with differential stem-like cell characteristics, such as basal gene expression of breast cancer or

CD133-positive versus glioma cells, to query the Cmap database for drugs that reverse the CSC-associated signatures.

(bottom) Suvà et al. (2009) [24] found that EZH2 plays a crucial role in tissue-specific stem-like cell maintenance and is overexpressed in GBM. Treatment with DZNep strongly impairs GBM CSC self-renewal *in vitro* and tumor-initiating capacity *in vivo*. The expression signature was established using CSC enriched gliospheroids from primary GBM cell cultures, with or without DZNep treatment.

Supplementary information, Table S6. Up-regulated and down-regulated proteins in the microwestern assay.

Up-regulation

Protein	Samle	Fold change
p-AMPKα (Thr172) (D79.5E) rabbit mAb	Control	**1.000**
	Thioridazine 5 uM	**1.287**
	Thioridazine 10 uM	**1.620**
	Thioridazine 15 uM	**3.403**
PTEN	Control	**1.000**
	Thioridazine 5 uM	**1.861**
	Thioridazine 10 uM	**6.263**
	Thioridazine 15 uM	**17.899**
p-PAK1 (Ser199/Ser204)	Control	**1.000**
	Thioridazine 5 uM	**1.075**
	Thioridazine 10 uM	**1.033**
	Thioridazine 15 uM	**3.823**

Down-regulation

Protein	Samle	Fold change
Bax	Control	**1.000**
	Thioridazine 5 uM	**0.869**
	Thioridazine 10 uM	**0.572**
	Thioridazine 15 uM	**0.682**
c-Myc	Control	**1.000**
	Thioridazine 5 uM	**0.978**
	Thioridazine 10 uM	**0.710**
	Thioridazine 15 uM	**0.464**
GSK-3β	Control	**1.000**
	Thioridazine 5 uM	**0.800**
	Thioridazine 10 uM	**0.505**
	Thioridazine 15 uM	**0.576**
p38α MAPK	Control	**1.000**
	Thioridazine 5 uM	**0.903**
	Thioridazine 10 uM	**0.598**
	Thioridazine 15 uM	**0.596**
p70 S6 kinase	Control	**1.000**
	Thioridazine 5 uM	**0.866**

	Thioridazine 10 uM	**0.496**
	Thioridazine 15 uM	**0.515**
p-acetyl CoA carboxylase	Control	**1.000**
	Thioridazine 5 uM	**0.598**
	Thioridazine 10 uM	**0.395**
	Thioridazine 15 uM	**0.414**
p-JNK (Thr183/Tyr185, Thr221/Tyr223)	Control	**1.000**
	Thioridazine 5 uM	**0.640**
	Thioridazine 10 uM	**0.244**
	Thioridazine 15 uM	**0.373**
p-mTOR (Ser2481)	Control	**1.000**
	Thioridazine 5 uM	**0.850**
	Thioridazine 10 uM	**0.409**
	Thioridazine 15 uM	**0.576**
Akt1/PKBα	Control	**1.000**
	Thioridazine 5 uM	**0.785**
	Thioridazine 10 uM	**0.471**
	Thioridazine 15 uM	**0.432**
AMPKα1	Control	**1.000**
	Thioridazine 5 uM	**0.661**
	Thioridazine 10 uM	**0.512**
	Thioridazine 15 uM	**0.577**
Cyclin B1	Control	**1.000**
	Thioridazine 5 uM	**0.801**
	Thioridazine 10 uM	**0.440**
	Thioridazine 15 uM	**0.433**
Cyclin E1	Control	**1.000**
	Thioridazine 5 uM	**0.746**
	Thioridazine 10 uM	**0.452**
	Thioridazine 15 uM	**0.420**
Cyclin E2	Control	**1.000**
	Thioridazine 5 uM	**0.724**
	Thioridazine 10 uM	**0.467**
	Thioridazine 15 uM	**0.216**
GRB2	Control	**1.000**

	Thioridazine 5 uM	**0.980**
	Thioridazine 10 uM	**0.691**
	Thioridazine 15 uM	**0.581**
HDAC3	Control	**1.000**
	Thioridazine 5 uM	**0.977**
	Thioridazine 10 uM	**0.673**
	Thioridazine 15 uM	**0.602**
NF- B p50	Control	**1.000**
	Thioridazine 5 uM	**0.835**
	Thioridazine 10 uM	**0.668**
	Thioridazine 15 uM	**0.639**
p-p70 S6 kinase (Thr389)	Control	**1.000**
	Thioridazine 5 uM	**0.865**
	Thioridazine 10 uM	**0.451**
	Thioridazine 15 uM	**0.462**
p21/WAF1/Cip1	Control	**1.000**
	Thioridazine 5 uM	**0.464**
	Thioridazine 10 uM	**0.205**
	Thioridazine 15 uM	**0.312**
Bcl2	Control	**1.000**
	Thioridazine 5 uM	**0.989**
	Thioridazine 10 uM	**0.661**
	Thioridazine 15 uM	**0.646**
EGFR	Control	**1.000**
	Thioridazine 5 uM	**0.905**
	Thioridazine 10 uM	**0.727**
	Thioridazine 15 uM	**0.730**
Jak2	Control	**1.000**
	Thioridazine 5 uM	**0.602**
	Thioridazine 10 uM	**0.241**
	Thioridazine 15 uM	**0.334**
p-Tuberin/TSC2	Control	**1.000**
	Thioridazine 5 uM	**0.680**
	Thioridazine 10 uM	**0.467**
	Thioridazine 15 uM	**0.574**

p-STAT6	Control	**1.000**
	Thioridazine 5 uM	**0.769**
	Thioridazine 10 uM	**0.649**
	Thioridazine 15 uM	**0.747**
Cyclin D1	Control	**1.000**
	Thioridazine 5 uM	**0.847**
	Thioridazine 10 uM	**0.693**
	Thioridazine 15 uM	**0.562**
p-EGFR (Tyr1086)	Control	**1.000**
	Thioridazine 5 uM	**0.670**
	Thioridazine 10 uM	**0.455**
	Thioridazine 15 uM	**0.554**
p-estrogen receptor α (Ser167)	Control	**1.000**
	Thioridazine 5 uM	**0.571**
	Thioridazine 10 uM	**0.422**
	Thioridazine 15 uM	**0.592**
p-PDGF receptor α (Tyr1018)	Control	**1.000**
	Thioridazine 5 uM	**0.914**
	Thioridazine 10 uM	**0.454**
	Thioridazine 15 uM	**0.539**
p-Raptor (Ser792)	Control	**1.000**
	Thioridazine 5 uM	**0.886**
	Thioridazine 10 uM	**0.600**
	Thioridazine 15 uM	**0.544**
p-Met (Tyr1003)	Control	**1.000**
	Thioridazine 5 uM	**0.744**
	Thioridazine 10 uM	**0.697**
	Thioridazine 15 uM	**0.630**
